# Upcycling of Waste Fluororubber to Photocurable High‐Performance Vinyl‐Terminated Liquid Fluororubber by Multifield Coupling One‐Pot Stepwise Reactions

**DOI:** 10.1002/advs.202501460

**Published:** 2025-05-28

**Authors:** Donghan Li, Lu Yu, Shurui Ning, Ping Li, Changle Chen, Dawei Zhao, Mingyi Liao, Qingshi Meng, Shixin Zhang, Qinghong Fang, Hailan Kang, Long Li, Jia Yang

**Affiliations:** ^1^ College of Materials Science and Engineering Shenyang University of Chemical Technology Shenyang 110142 China; ^2^ Liaoning Key Laboratory of Polymer Materials Application Technology Shenyang University of Chemical Technology Shenyang 110142 China; ^3^ Key Laboratory of Precision and Intelligent Chemistry Department of Polymer Science and Engineering University of Science and Technology of China Hefei 230026 China; ^4^ Key Laboratory on Resources Chemicals and Materials of Ministry of Education Shenyang University of Chemical Technology Shenyang 110142 China; ^5^ College of Transportation Engineering Dalian Maritime University Dalian 116026 China; ^6^ College of Aerospace Engineering Shenyang Aerospace University Shenyang 110136 China; ^7^ College of Science Shenyang University of Chemical Technology Shenyang 110142 China

**Keywords:** multifield coupling, one‐pot step‐wise, vinyl‐terminated liquid fluororubbers, upcycling, waste fluororubbers

## Abstract

To address the challenges of recycling and high‐value utilization of waste fluororubbers, an effective method is reported for producing novel photocurable vinyl‐terminated liquid fluororubbers (VTLF) with elevated fluorine content (63.1%), superior temperature resistance (*T*_10%_ = 335 °C) from commercial waste fluororubbers. The approach employs a streamlined, multifaceted system (oxidative degradation/condensation reaction) integrating microwave, mechanical, and steady‐state temperature fields. This system facilitates both efficient recycling and high‐value transformation of waste fluororubbers. Initially, waste fluororubbers undergo controlled/oxidative degradation induced by alkali and hydrogen peroxide to yield carboxyl‐terminated liquid fluororubbers (CTLF). Subsequently, condensation reaction system efficiently converts carboxyl groups into photoreactive vinyl groups. Ultimately, environmentally friendly and efficient photocuring of VTLF is achieved. The nonthermal effects of microwave fields reduce the total process time to just 1 h. The resulting photocured VTLF exhibits not only the comprehensive properties of conventional fluororubbers but also excellent chemical stability and unique light transmittance (94.21%). This study proposes a green, straightforward upcycling strategy within the circular economy framework to mitigate environmental issues associated with rubber's covalent crosslinking. Furthermore, it opens avenues for designing and synthesizing novel fluoropolymers for diverse applications.

## Graphical abstract

Herein, waste fluororubbers undergo controlled oxidative degradation induced by alkali and hydrogen peroxide to yield carboxyl terminated liquid fluororubbers. Subsequently, condensation reaction system efficiently converts carboxyl groups into photoreactive vinyl groups. Ultimately, an environmentally friendly and efficient photocuring of vinyl‐terminated liquid fluororubbers is achieved, which is of great significance for the global recycling of waste fluororubbers.

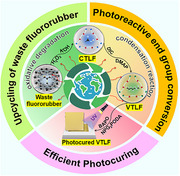

## 1 Introduction

The manufacture of rubber products invariably involves the vulcanization of elastomer molecules, forming stable 3D crosslinked networks that confer elasticity and strength. However, these networks are typically non‐recyclable and non‐biodegradable.^[^ ^1^, ^2^, ^3^ ^]^ Conventional disposal methods such as landfills and incineration not only directly harm the environment but also pose potential risks to human health.^[^ ^4^, ^5^, ^6^ ^]^

As the most attractive special‐purposed synthetic rubber, due to the robust C─F bond with a high bond energy of 485 kJ mol^−1^ and the shielding effect of the fluorine atom on the C─C bond in the molecular structure of fluororubbers. This imparts excellent thermal stability, chemical resistance, and oxidation resistance.^[^ ^7^, ^8^, ^9^ ^]^ However, the excellent stability also leads to the natural degradation and high value utilization of waste fluororubbers is very difficult.^[^ ^10^, ^11^, ^12^ ^]^ Therefore, for the recycling of waste fluororubber is an urgent problem, which is of great significance for the sustainable development of the ecological environment.

In view of the rapid development of industries such as new energy, aerospace, medical protection, additive manufacturing, and flexible sensors, functional fluoropolymers can be found everywhere and the functionalization of this field has attracted a lot of attention because of their excellent stability, surface properties, and unique mobility. Consequently, the synthesis of highly fluorine‐containing reactive polymers with superior overall properties has garnered significant attention.^[^ ^9^, ^13^, ^14^, ^15^ ^]^ Nevertheless, the production of fluoropolymers with a high content of fluorine proves to be challenging and intricate. Currently, the predominant synthetic techniques encompass the functional initiator method,^[^ ^16^ ^]^ stepwise polymerization,^[^ ^17^, ^19^ ^]^ iodine transfer polymerization (ITP),^[^ ^20^ ^]^ atom transfer radical polymerization (ATRP),^[^ ^18^, ^21^ ^]^ and reversible addition‐fragmentation chain transfer polymerization (RAFT).^[^ ^22^ ^]^ However, concerns about the potential hazards of perfluorinated and polyfluoroalkyl substances (PFAS) globally, how to reduce the residual PFAS in the system has become a new challenge for the “green” preparation of high fluoropolymers.^[^ ^23^, ^24^, ^25^ ^]^

So if a groundbreaking “Upcycling” method is devised, it will not merely facilitate the direct and efficient utilization of waste fluororubbers, but also cater to the need for reactive fluoropolymers with superior overall properties.^[^ ^26^ ^]^ This innovation is bound to hold significant theoretical implications and practical worth.

Fortunately, the oxidative degradation method, utilizing the chemical properties of the vinylidene fluoride structure in fluororubbers, offers an alternative pathway for synthesizing highly fluorine‐containing reactive polymers, as the product is CTLF and does not require additional perfluoro and PFAS.^[^ ^27^, ^28^ ^]^ Recently, Li et al. conducted a comprehensive investigation into the mechanism of the dehydrofluorination, rearrangement, and oxidative degradation reactions of fluororubbers containing vinylidene fluoride structures under alkaline conditions.^[^ ^29^, ^30^ ^]^ Typically, the synthesis of CTLF is marked by low efficiency and challenging control due to prolonged reaction times (24 h) and low reaction temperatures (‐5–10 °C). Various strategies, including the employment of hydroxyl‐ and siloxyl‐functionalized liquid fluororubbers and isocyanate‐terminated liquid fluororubbers,^[^ ^31^, ^32^, ^33^, ^34^ ^]^ microwave‐assisted oxidative degradation have been explored to enhance curing efficiency and overall performance.^[^ ^35^, ^36^ ^]^

Herein, aiming for the upcycling of waste fluororubbers, a novel approach involving multi‐field coupling of one‐pot stepwise reactions has been developed. This method facilitates the efficient synthesis of high‐performance photocurable liquid fluoropolymers with high fluorine content, alongside controllable molecular weight and end group content. The relationships between the chain structure and the properties of the raw materials and products were meticulously studied, and the reaction mechanisms of each phase were elucidated. This research is of great significance for the global recycling of used fluororubbers and provides a new idea and reference for the design and synthesis of functional fluoropolymers.

## 2 Results and Discussion

**Figure 1:**
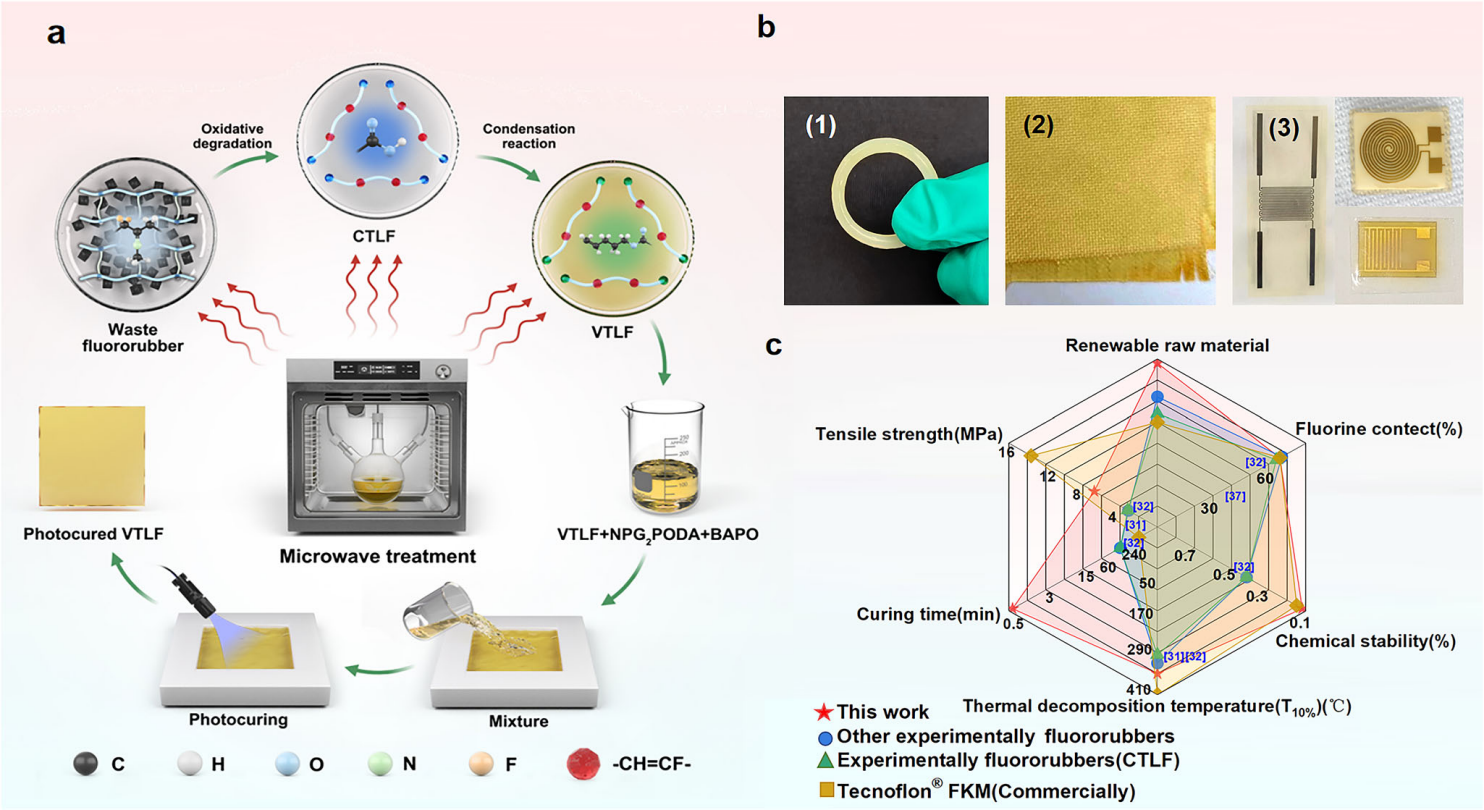
a) The synthetic routes for photocured VTLF. b) (1) 3D printed VTLF seals. (2) Photocured VTLF/aramid fiber reinforced composite. (3) Photocured VTLF as a substrate for flexible sensors. c) Comparison of the properties of this work with other fluororubbers.^[^ ^31^, ^32^, ^37^ ^]^

A photocrosslinking system for fluororubbers (**Figure** **1**a) was created through a two‐step process. In the first step, waste fluororubbers underwent oxidative degradation to synthesize CTLF efficiently under multi‐field coupling conditions. CTLF then underwent a condensation reaction with 4‐penten‐1‐ol or 3‐buten‐1‐ol, introducing vinyl groups at the molecular chain ends. In the second step, a crosslinked network of high‐performance fluorinated materials was formed via UV‐initiated free radical polymerization. Experimental conditions and product structures of both the oxidative degradation and condensation reactions were analyzed to determine optimal reaction conditions and clarify reaction mechanisms. Figure 1b illustrates the 3D printed VTLF seals, photocured VTLF/aramid fiber reinforced composite and photocured VTLF as a substrate for flexible sensors. Figure 1c compares the properties of this work with other high‐performance materials, upcycling fluororubber demonstrate excellent all‐round performance.

### 2.1 Synthesis of CTLF

Multi‐field coupling, incorporating microwave, mechanical, and steady‐state temperature fields, facilitated the rapid oxidative degradation of waste fluororubbers to efficiently produce CTLF. Potassium hydroxide (KOH), hydrogen peroxide (H_2_O_2_) and benzyltriethylammonium chloride (BTEAC) were applied to decompose commercially available fluororubbers (refer to Table S1, Supporting Information for the composition of waste fluororubber).

Under alkaline and oxidative conditions, the degradation of high‐molecular‐weight fluoropolymers containing vinylidene fluoride structures proceeds in two distinct stages (Figure S1, Supporting Information). Initially, OH^−^ ions from KOH attack the ─H─ in ─CF_2_CH_2_─, causing dehydrofluorination and rearrangement reactions. This autocatalytic process forms fluorine‐containing double bond (─C═C─), facilitating reactions among adjacent groups. These chain fragments rearrange to create multiple sequence structure double bonds, adhering to Zaitsev's and Hofmann's rules. In the second stage, oxidants target the chain's double bonds, initiating oxidative degradation reactions. This process breaks down the lengthy molecular chains and crosslinking networks of high‐molecular‐weight fluoropolymers, resulting in linear telechelic fluoropolymers with carboxyl groups at both chain ends (Figures S1 and S2, Supporting Information). Despite vulcanized fluororubbers has excellent chemical and thermal stability, recycling vulcanized fluoropolymers remain challenging due to the difficulty in conducting oxidative degradation of fluoropolymers.

In addition, the oxidative degradation reaction system of waste fluororubbers with inorganic bases and hydrogen peroxide represents a typical heterogeneous reaction system. Without phase transfer catalysis, the interaction between OH^−^ and H_2_O_2_ with the molecular chains of fluororubbers is ineffective, leading to dehydrogenation and oxidative degradation reactions proceeding at a very slow rate. Herein, based on the non‐thermal effects of microwave radiation, microwaves accelerate the ionization of KOH and H_2_O_2_, enhancing the production of OH^−^ and O^−^ free radicals. The absorption of microwave energy increases the collision frequency of polar groups such as OH^−^, O^−^, and F^−^, thereby accelerating dehydrofluorination, rearrangement, and oxidative degradation reactions in fluororubbers.^[^ ^29^, ^30^ ^]^ Concurrently, the non‐thermal effects of microwave radiation elevate the average energy of molecules in the reaction system, promoting the movement of fluororubber molecular chains and reducing the reaction's activation energy to enhance reaction efficiency.

**Figure 2:**
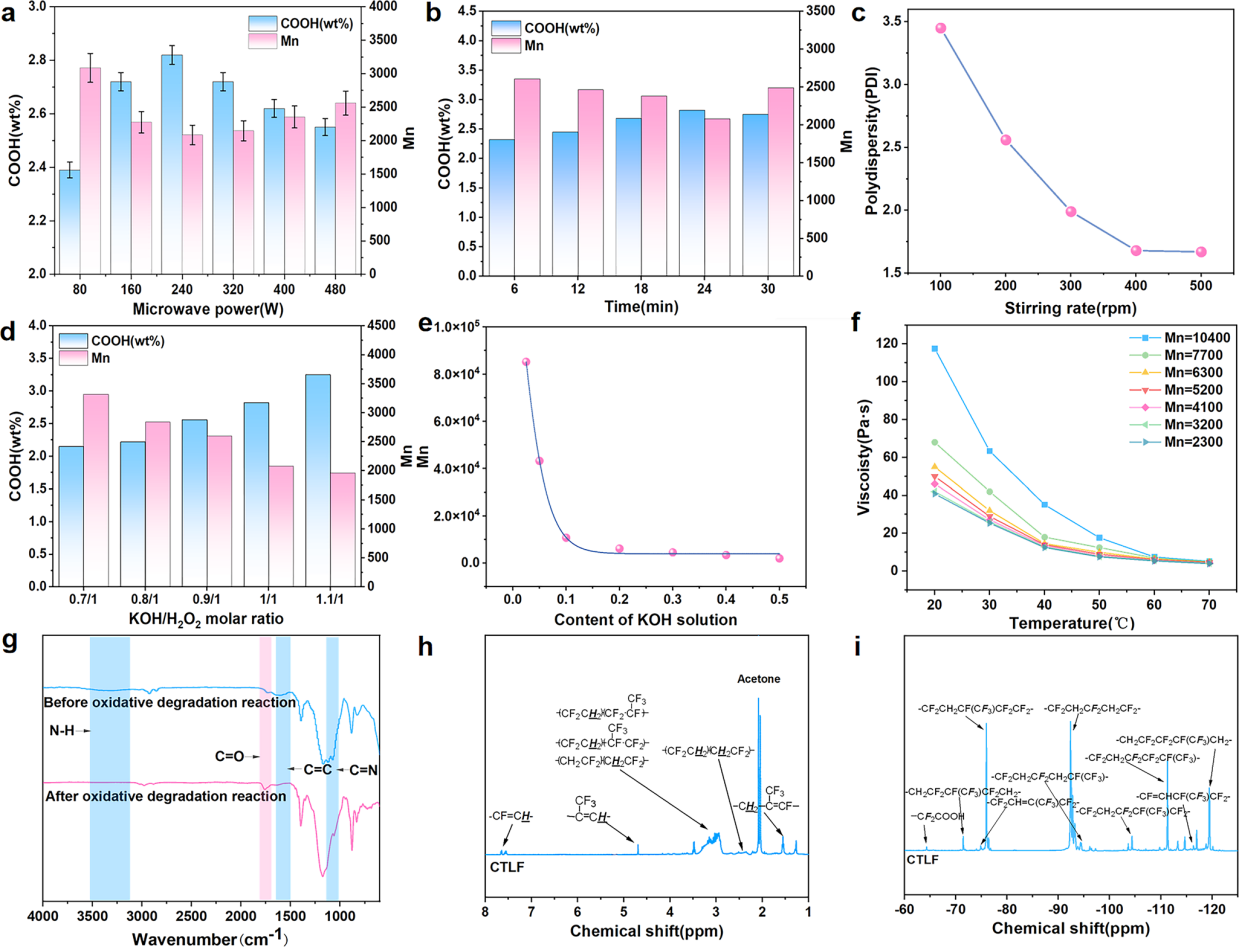
a) Effect of microwave power on the oxidative degradation reaction of waste fluororubbers when in acetone at 10 °C with microwave radiation time of 24 min and KOH/H_2_O_2_ = 1.0/1.0. b) Effect of microwave radiation time on the oxidative degradation reaction of waste fluororubbers when in acetone at 10 °C with microwave power of 240 W and KOH/H_2_O_2_ = 1.0/1.0. c) Effect of stirring rate on the oxidative degradation reaction of waste fluororubbers. d) Effect of KOH/H_2_O_2_ molar ratio on the oxidative degradation reaction of waste fluororubbers. e) Effect of KOH solution on the oxidative degradation reaction of waste fluororubbers. f) Dynamic viscosity of CTLFs with an increase of temperature. g) FT‐IR spectra of waste fluororubbers before and after oxidative degradation reaction. h) ^1^H‐NMR spectra of CTLF. i) ^19^F‐NMR spectra of CTLF.

To achieve efficient and controlled oxidative degradation of waste fluororubbers while minimizing side reactions like crosslinking, hydroxyaldehyde condensation, and excessive degradation, we employed a temperature control system in a microwave reactor and established a coupled external field comprising a microwave field, mechanical stirring, and steady‐state temperature control. Initially, we systematically investigated the effects of microwave radiation time and power on the oxidative degradation of fluororubbers. Our findings indicate that lower microwave power transfers less energy to the reaction system, resulting in incomplete dehydrofluorination and oxidative degradation of the ─C═C─. Conversely, excessive microwave power leads to rapid ionization of KOH and H_2_O_2_, causing H_2_O_2_ decomposition into H_2_O and O_2_, thereby reducing oxidation capacity and yielding CTLF with higher molecular weights and lower carboxyl group contents (**Figure** **2**a and Table S3, Supporting Information). Fortunately, microwave radiation demonstrated an ideal catalytic effect. As illustrated in Figure 2b and Table S4 (Supporting Information), even with a microwave irradiation time of just 6 min, fluoropolymer molecular weights reached about 2600 g mol^−1^. This represents a significant enhancement in reaction efficiency compared to traditional oxidative degradation methods, which typically require several hours.

Following this, the impact of mechanical stirring on the oxidative degradation reaction of waste fluororubbers was explored, and the outcomes are illustrated in Figure 2c. The stirring speed had a significant effect on the polymer dispersity index (PDI) of CTLF, which decreased as the stirring speed increased, stabilizing at 1.68 at 400 rpm. This phenomenon occurs because the oxidative degradation reaction process of waste fluororubbers involves intricate elimination and oxidative reactions, causing localized overheating and a wide PDI of the product. Higher stirring rate not only maintain stable reaction temperatures but also enhance reaction system uniformity, thereby lowering the PDI of the CTLF.

Furthermore, we investigated the effects of varying amounts of KOH and H_2_O_2_ on the carboxyl content and molecular weight of CTLF, as illustrated in Figure 2d and Table S5 (Supporting Information). Increasing the molar ratio of KOH/H_2_O_2_ resulted in higher carboxyl group content in CTLF, reaching up to 3.25 wt%. This increase is due to KOH generating more double bonds in the waste fluororubber chains, facilitating oxidation of broken bonds and thereby increasing carboxyl content while reducing molecular weight. However, excessively high KOH/H_2_O_2_ ratios led to lower molecular weights of CTLF, potentially impacting the properties of cured products. Optimal control over CTLF molecular weight was achieved at a 1.0:1.0 KOH/H_2_O_2_ rate, as shown in Figure 2e. Increasing KOH content from 0.03 to 0.5 decreased CTLF molecular weight from 85000 to 6000. Polar polymers generally exhibit strong chain interactions affecting processing characteristics such as viscosity and hardness, critical in product molding and application. Interestingly, CTLF prepared through oxidative degradation reaction demonstrated excellent mobility at room temperature, with dynamic viscosity decreasing as molecular weight decreased. At an Mn of about 2300, CTLF exhibited a low dynamic viscosity of 4 Pa s at 70 °C. Conversely, higher CTLF molecular weights (≈10400) significantly increased dynamic viscosity (118 Pa s at 20 °C), reducing system compatibility and increasing molding defects. Therefore, for optimal processing performance, CTLF with molecular weights below 7700 was preferred (Figure 2f).

The chain structure of the waste fluororubbers before and after oxidative degradation reaction under microwave radiation was confirmed by Fourier transform infrared (FT‐IR) spectroscopy. As shown in Figure 2g, the peaks at 880, 1079, and 1395 cm^−1^ were attributed to the stretching vibrations of ─CF─, ─CF_2_─ and ─CF_3_, respectively. The peaks at 2968 and 2921 cm^−1^ are assigned to the ─C─H─ symmetric stretching vibration and the antisymmetric stretching vibration of methylene groups (─CH_2_─), respectively, and the peaks at 3357 and 1650 cm^−1^ are assigned to the ─NH─ and ─C═C─ stretching vibrations, respectively. Upon comparison with the FT‐IR spectrum of waste fluororubbers, following oxidative degradation reaction, the ─NH─ peak at 3357 cm^−1^ vanished, the ─C═C─ peak at 1685 cm^−1^ notably attenuated, and a new peak attributable to ─C═O carboxyl groups (─CF_2_COOH) emerged at 1767 cm^−1^. This provides evidence of the successful oxidative degradation reaction and the introduction of carboxyl groups at the molecular chain ends.

The structure of CTLF was characterized using ^1^H nuclear magnetic resonance (^1^H‐NMR), depicted in Figure 2h. The ^1^H‐NMR spectra reveal multiplets ranging from 2.85 to 3.51 ppm, attributed to methylene structures of ─CH_2_CF_2_─CH_2_CF_2_─, ─CF_2_CH_2_─CF_2_CF(CF_3_)─, and ─CF_2_CH_2_─CF(CF_3_)CF_2_─ (head‐to‐tail units). Peaks between 2.28 and 2.62 ppm correspond to ─CF_2_CH_2_─CH_2_CF_2_─ structures (head‐to‐head units). Additionally, peaks at 1.78 and 4.69 ppm denote structures of ─CH_2_─(CF_3_)C═CF─ and ─(CF_3_)C═CH─ formed via elimination reactions following Zaitsev's rule, respectively. Peaks within the range of 7.52 to 7.69 ppm represent ─CH═CF─ structures formed following Hofmann's rule.

The structures of CTLF were further confirmed by ^19^F‐NMR spectroscopy. As shown in Figure 2i, signals corresponding to ─CF_3_ in HFP were observed in the range of ‐70 to ‐82 ppm. The signals corresponding to ─CF_2_─ in VDF were observed in the range of ‐92 to ‐117 ppm. The signal at −119.43 ppm corresponded to ─CF_2_─ in HFP. Furthermore, a novel signal corresponding to _—CF_2_—adjacent to terminal carboxyl groups was identified at ‐64.35 ppm. This observation of the ─CF_2_COOH structure aligns with the FT‐IR results, confirming the successful synthesis of CTLF. The fluorine contents of CTLF were also calculated, with specific characteristic peaks outlined in Table S2 (Supporting Information). The characteristic peak area for the VDF sequence structure was defined as ∑CF_2_ = *I*_‐64.30_+*I*_‐92.36_+*I*_‐94.38_+*I*_‐96.14_+*I*_‐104.31_+*I*_‐109.69_+*I*_‐111.27_+*I*_‐113.29_+*I*_‐114.78_+*I*_‐116.24_, and for the HFP sequence structure as ∑CF_3_ = *I*_‐71.35_+*I*_‐74.85_+*I*_‐75.88_+*I*_‐81.24_+*I*_‐81.48_. The monomer content (X) of CTLF was determined using the following equations.

(1) $$X_{\mathrm{VDF}}=\frac{\sum {\mathrm{CF}}_{2}}{2/3\sum {\mathrm{CF}}_{3}+\sum {\mathrm{CF}}_{2}}\;\times 100\%$$

(2) $$X_{\mathrm{HFP}}=\frac{2/3\sum {\mathrm{CF}}_{3}}{2/3\sum {\mathrm{CF}}_{3}+\sum {\mathrm{CF}}_{2}}\;\times 100\%$$

The calculation resulted in *X*_VDF_ and *X*_HFP_, and the fluorine content (*X*_F_) in the sample was computed using the following equation:

(3) $$X_{F}=\frac{X_{\mathrm{VDF}}\times 38.04+X_{\mathrm{HFP}}\times 114.02}{X_{\mathrm{VDF}}\times 64.04+X_{\mathrm{HFP}}\times 150.02}\;\times 100\%$$

After calculation, the fluorine content in the CTLF was 64.6%.

### 2.2 Synthesis of VTLF

**Figure 3:**
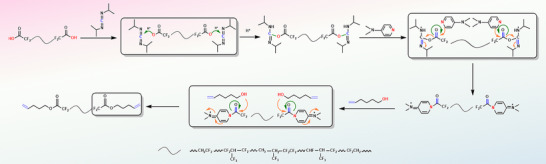
Mechanism of preparation of VTLF by condensation reaction.

Based on the controllable preparation of CTLF, a carboxyalkenylation reaction system was established for these highly polar fluoropolymers. Through design and optimization, the continuous reaction and synthesis of waste fluororubbers‐CTLF‐VTLF can be achieved, and the introduction of the photoreactive vinyl group at the end of the reactive fluoropolymer molecular chain to synthesize the photocurable VTLF is enabled. This approach provides a new reference for the designing and synthesizing of high‐performance fluoropolymer precursors. The reaction involves condensation between carboxyl and hydroxyl groups (**Figure** **3**) which can also be conducted under multi‐field coupling conditions, with 4‐penten‐1‐ol and 3‐buten‐1‐ol selected as the vinyl‐containing compounds.

During the investigation of the reaction system, it was observed that dicyclohexylcarbodiimide (DCC) produces white needle‐like N, N‐dicyclohexylurea, which can readily mix with viscous liquid fluororubbers, posing challenges in removal and significantly impacting product purity. Systematic screening identified N, N´‐diisopropylcarbodiimide (DIC) as a superior alternative in the fluoropolymer reaction system due to its excellent reactivity. Additionally, the byproduct (diisopropylurea) formed during the reaction exhibits good solubility in organic solvents, facilitating easier removal. Consequently, DIC was chosen as the condensation agent instead of DCC.

**Figure 4:**
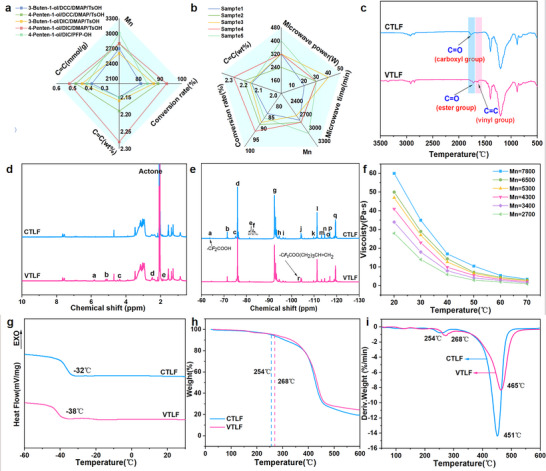
a) Effect of different systems on the conversion rate of VTLF. b) Effect of different reaction conditions on VTLF structure. c) FT‐IR spectra of CTLF and VTLF. d) ^1^H‐NMR spectra of CTLF and VTLF. e) ^19^F‐NMR spectra of CTLF and VTLF. f) Dynamic viscosity of VTLFs with an increase of temperature. g) DSC curves of CTLF and VTLF. h) TGA thermograms of CTLF and VTLF. i) DTG thermograms of CTLF and VTLF.

The COOH in CTLF reacts with DIC to form acyl isourea, which exhibits higher reactivity than carboxylic acid. Acyl isourea can undergo rearrangement to generate diisopropylurea, a byproduct with good solubility in typical organic solvents, facilitating easy removal. In the presence of P‐toluenesulfonic acid (TsOH) as a protonated acid, COOH in CTLF is protonated, promoting nucleophilic reaction with 4‐penten‐1‐ol. The resulting acyl pyridine is subsequently attacked by 4‐penten‐1‐ol to yield VTLF. Furthermore, we observed a higher percentage of carboxyl groups when using 4‐penten‐1‐ol (**Figure** **4**a). This may be attributed to the additional methyl group in 4‐penten‐1‐ol compared to 3‐buten‐1‐ol, wherein the electron‐donating effect of the methyl group disperses electron density away from the oxygen atom. As a result, the hydrogen atom binding capacity weakens, facilitating easier dissociation and thereby promoting the reaction.

Furthermore, the influence of microwave power and time on the condensation reaction was investigated, and the results are depicted in Figure 4b and Tables S6 and S7 (Supporting Information). The most significant enhancement in reaction efficacy was observed at a microwave power of 320 W. At a microwave exposure time of 20 min, the conversion rate of COOH in CTLF was about 85%, which increased to 93% with prolonged exposure time.

Under optimal reaction conditions (COOH/4‐penten‐1‐ol/DIC/DMAP/TsOH = 1.00/2.00/1.00/0.15/0.10) (Tables S8–S11, Supporting Information), the FT‐IR spectra of CTLF and VTLF are presented in Figure 4c. Following the condensation reaction, the intensity of the characteristic peak at 1767 cm^−1^ assigned to the ─COOH groups of the liquid fluororubbers notably decreased. New peaks emerged at 1722 cm^−1^ and 1647 cm^−1^, attributed to the ─C═O of the ester group and the ─C═C─ of the vinyl group, respectively. Confirmation of these changes came from ^1^H‐NMR and ^19^F‐NMR spectra (Figure 4d,e). In Figure 4d, multiple peaks around 5.82 ppm and 5.13 ppm corresponded to the ─CH═CH_2_ structure. Figure 4e indicated that post‐condensation, the characteristic peak of ─CF_2_COOH decreased, while the characteristic peak of ─CF_2_COO(CH_2_)_3_CH═CH_2_ appeared at ‐103.10 ppm, confirming the success of the condensation reaction. Additionally, most signals in these spectra closely resembled those in the CTLF spectra, demonstrating the reaction system's excellent selectivity without compromising the backbone structure of the fluoropolymers. The fluorine content of the VTLF was further calculated using Equations ((1), (2), (3)), revealing a fluorine content of 63.1%.

The dynamic viscosity of the VTLFs was examined and is illustrated in Figure 4f. Conversion of the molecular chain end to a more flexible segment with reduced polarity enhanced the fluidity of VTLF at equivalent temperatures relative to CTLF. Specifically, at a molecular weight of about 2700, VTLF exhibited a dynamic viscosity of 28 Pa s at 20 °C and 1 Pa s at 70 °C. The thermal properties of CTLF and VTLF were analyzed using differential scanning calorimetry (DSC) and thermogravimetric analysis (TGA), as shown in Figure 4g–i.

Regarding low‐temperature properties, CTLF and VTLF exhibited lower glass transition temperatures (*T*_g_) −32 °C and −38 °C due to their shorter molecular chain lengths and lower molecular weights, respectively. VTLF, with its flexible chain end segment of weaker polarity, demonstrated improved low‐temperature properties compared to CTLF, enhancing their processing and molding capabilities at lower temperatures. In terms of high‐temperature stability, both CTLF and VTLF showed robust thermal stability thanks to their similar molecular chain structures to fluororubbers. The thermal decomposition temperatures (*T*_5%_) of CTLF and VTLF were 254 °C and 268 °C, *T*_10%_ of CTLF and VTLF were 308 °C and 335 °C.

In summary, through the implementation of multi‐field coupling reaction reinforcement technology, we successfully synthesized CTLF and VTLF with superior comprehensive performance, controllable molecular chain structure, and molecular weight. This approach effectively addresses the challenge of balancing the strong polarity and low viscosity typically associated with fluoropolymers.

### 2.3 Photocuring of the VTLF

**Figure 5:**
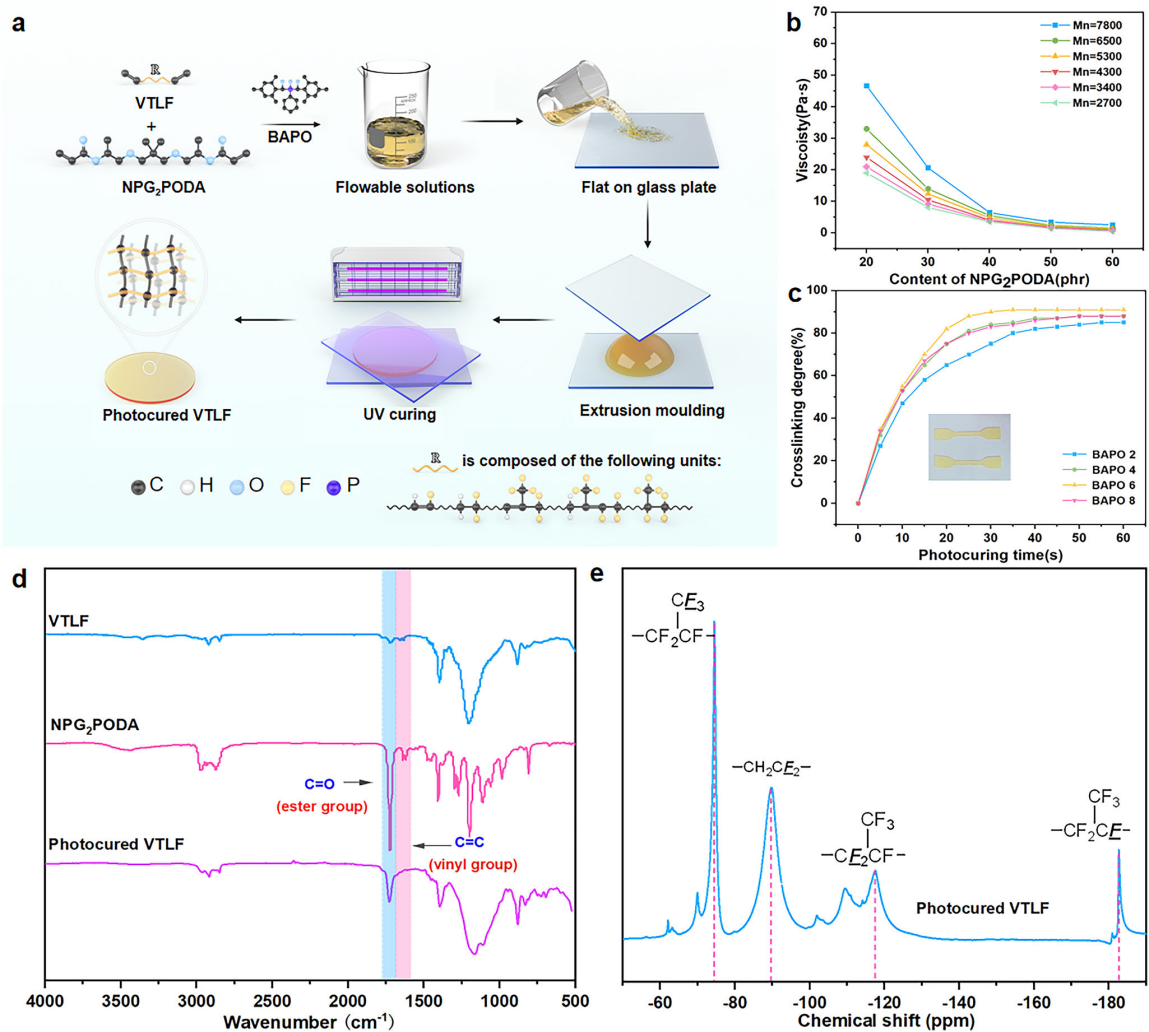
a) Mechanism of the VTLF photocuring reaction. b) Dynamic viscosity of VTLF with increasing NPG_2_PODA content. c) Degree of crosslinking of VTLF with increasing photocuring time. d) FT‐IR spectra of VTLF, NPG_2_PODA, and the photocured VTLF. e) ^19^F‐SSNMR spectra of a photocured VTLF.

Drawing upon the photochemical reactivity of the vinyl group, we established a radical UV‐curing system for VTLF and clarified its underlying reaction mechanism. Additionally, we investigated how the molecular chain structure influences the properties of the resulting photocured VTLFs. In this study, bifunctional polyoxy(methyl‐1,2‐ethanediyl) (NPG_2_PODA) was selected as the active diluent, and phenylbis(2,4,6‐trimethylbenzoyl) phosphine oxide (BAPO) was used as the photoinitiator. **Figure** **5**a illustrates the molding process involved in producing photocured VTLF.

The curing process of VTLF involves two distinct steps. Initially, under UV light at 405 nm, BAPO undergoes photolysis to yield two trimethylbenzoyl groups and one phenylphosphoyl group (three active free radicals). These radicals initiate the generation of monomeric free radicals from VTLF and NPG_2_PODA. Subsequently, a crosslinked network forms between VTLF and NPG_2_PODA through radical polymerization initiated by these free radicals. Importantly, the UV‐curing molding process does not involve the use of solvents; instead, it relies solely on the excellent fluidity of VTLF and ultraviolet irradiation to achieve rapid and room temperature formation of a fluororubber‐like material.

According to the literature, the viscosity of a polymer UV‐curing system plays a crucial role in the degree of crosslinking and the quality of molded photocured samples.^[^ ^38^ ^]^ High viscosity can impede UV light penetration during curing, resulting in incomplete reactions, and increase resistance to flow in the precursor, leading to uneven product quality. Therefore, we investigated how the dynamic viscosity of the system is affected by varying NPG_2_PODA content. Figure 5b illustrates that increasing NPG_2_PODA content significantly improves system fluidity. Beyond 50 phr, the viscosity flattens out, reaching a minimum of 0.5 Pa s at 60 °C, indicating an optimal NPG_2_PODA content of 60 phr.

The rapid photolysis of photoinitiators is known to generate high concentrations of active radicals, crucial for accelerating the rate of photopolymerization. However, excessive concentrations can prematurely terminate photopolymerization due to competitive radical–radical coupling reactions. Therefore, maintaining moderate photoinitiator concentration is key to enhancing photopolymerization rate and curing efficiency. Conversely, an excessive concentration of photoinitiators does not guarantee higher curing efficiency or crosslinking density and may lead to network defects due to residual initiators. Figure 5c demonstrates that BAPO effectively initiates radical crosslinking reactions of vinyl groups in VTLF under UV conditions, with curing efficiency increasing with higher BAPO content. At 6 phr BAPO, VTLF achieved a reaction efficiency of up to 30 s.

The chemical structures of VTLF, NPG_2_PODA, and photocured VTLF were characterized by FT‐IR, as depicted in Figure 5d. Compared with VTLF, the peak strength of the vinyl group at 1643 cm^−1^ in the photocured VTLF was significantly reduced, and a peak corresponding to the ester group emerged at 1722 cm^−1^, indicating radical photocuring and crosslinking of VTLF in this system. To verify these findings, the photocured VTLF structure was further characterized using ^19^F‐SSNMR, as shown in Figure 5e. Additionally, the fluorine content of the photocured VTLFs was calculated using Equations (1–, 3), where ∑CF_2_ represents the integral of the VDF sequence (∑CF_2_ = *I*_‐63.05_ + *I*_‐89.61_ + *I*_‐101.90_ + *I*_‐109.30_ + *I*_‐117.31_) and ∑CF_3_ represents the integral of the HFP sequence (∑CF_3_ = *I*_‐69.91_ + *I*_‐74.19_). The fluorine content of the photocured VTLF was determined to be about 60%, indicating that it retains a high fluorine content post‐photocuring.

**Figure 6:**
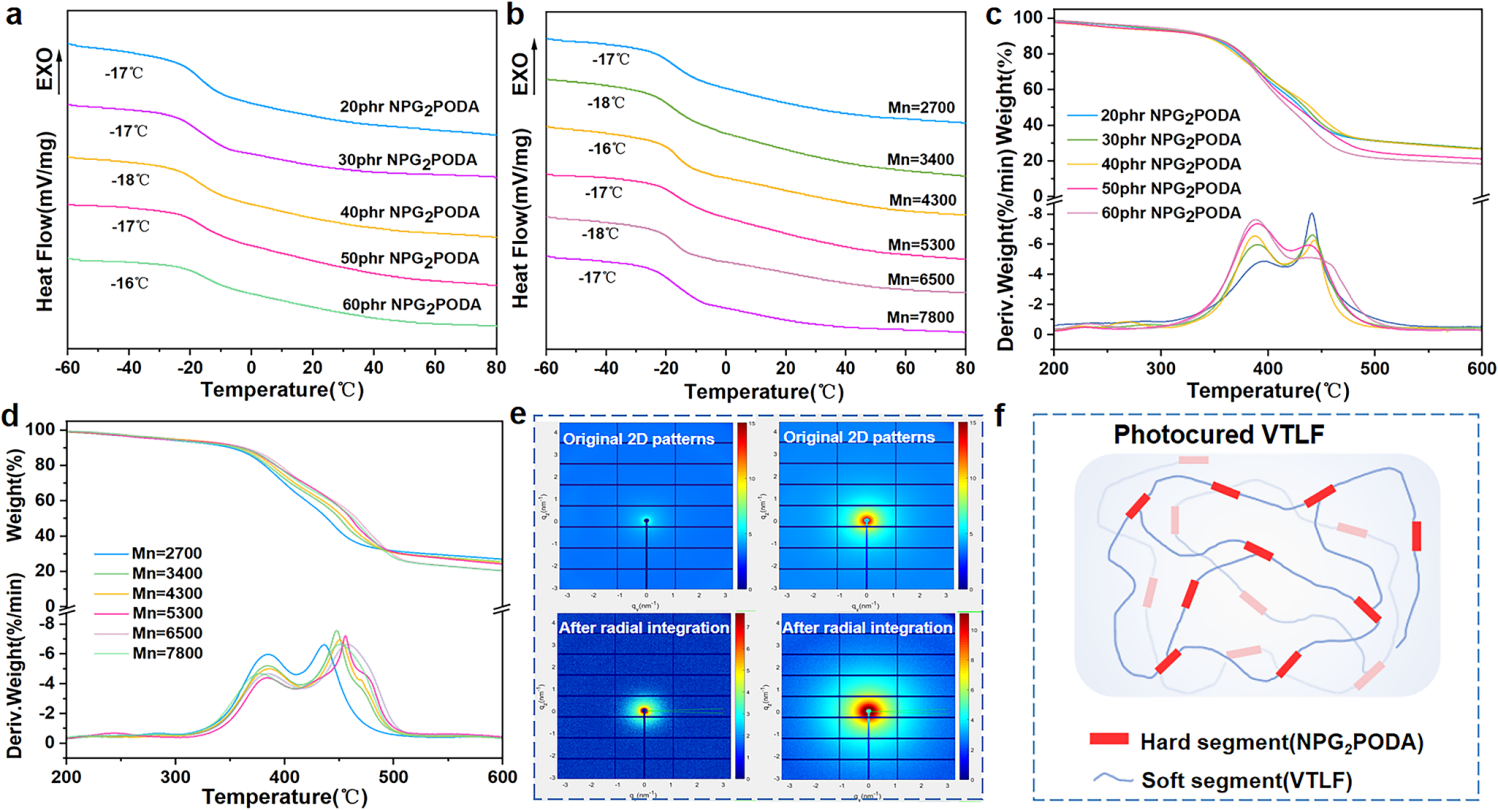
a) DSC curves of the photocured VTLFs at different NPG_2_PODA dosages. b) DSC curves of photocured VTLFs with different Mn values. c) TGA and DTG thermograms of VTLFs photocured with different NPG_2_PODA concentrations. d) TGA and DTG thermograms of photocured VTLFs with different Mn values. e) Synchrotron radiation 2D patterns of the fluororubbers film and photocured VTLF film. f) Schematic structure of the photocured VTLF.

Based on the structural characterization, the effects of the NPG_2_PODA content and the molecular weight of the VTLF on the properties of the photocured VTLF were systematically investigated. In addition, we also investigated the effect of the amount of photoinitiator BAPO on the photocuring time (Figure S3, Supporting Information), effect on mechanical properties of photocuring products (Figures S4 and S5, Supporting Information). In terms of thermal properties, the low‐temperature properties of the photocured VTLF were investigated by DSC, as shown in **Figure** **6**a,b. Since the crosslinked network structure was formed after the VTLF photocuring reaction, the *T*_g_ increased from ‐38 °C to about ‐17 °C compared with that of the uncrosslinked VTLF. Nevertheless, it is exciting that the low‐temperature properties of photocured VTLF are still superior to those of intact fluororubber (Figure S6, Supporting Information). Moreover, the results also showed that the content of NPG_2_PODA and the molecular weight of the VTLF had a limited influence on the *T*_g_ of the photocuring system. The results also showed that the influence of the NPG_2_PODA content and VTLF molecular weight on the *T*_g_ of the photocured products was limited due to the linear flexible molecular chain structure of the VTLF.

Then, the high‐temperature stability of the photocured VTLF was investigated by TGA, as shown in Figure 6c,d. The main thermal degradation of these materials occurred in the temperature range of 300–500 °C. The first stage occurred at 300–400 °C, and we speculate that this stage is mainly the decomposition of hard segments (NPG_2_PODA) in the molecular chain of the photocured VTLF. The second stage occurred at 400–500 °C and mainly involved the decomposition of soft segments (VTLF) in the molecular chain of the photocured VTLF. Moreover, with increasing molecular weight, the thermal stability of the photocured VTLF significantly improved; *T*_10%_ increased from 331 °C to 346 °C, while *T*_max_ increased from 439 °C to 463 °C.

To investigate the submicroscopic structure of the photocured VTLF. We performed synchrotron radiation measurements at the BL16b1 beamline of the SSRF with a low scattering vector *q*. The results of synchrotron radiation are presented in Figure 6e. Based on the 2D synchrotron radiation patterns of the fluororubber film and the photocured VTLF film, the fluororubber film exhibits almost no synchrotron radiation signal, whereas the photocured VTLF film demonstrates a strong synchrotron radiation signal (Figure 6e). This contrast is further illustrated (Figure S7, Supporting Information), where the scattering intensity of the photocured VTLF is significantly higher than that of the fluororubber. It is well known that the square of the sample density contrast affects the synchrotron radiation scattering intensity;^[^ ^39^ ^]^ thus, this higher synchrotron radiation scattering intensity of photocured VTLF may be due to phase separation. The photocured VTLF in this study exhibited a phase separation structure with excellent mechanical properties. This was consistent with the above TGA results.

**Figure 7:**
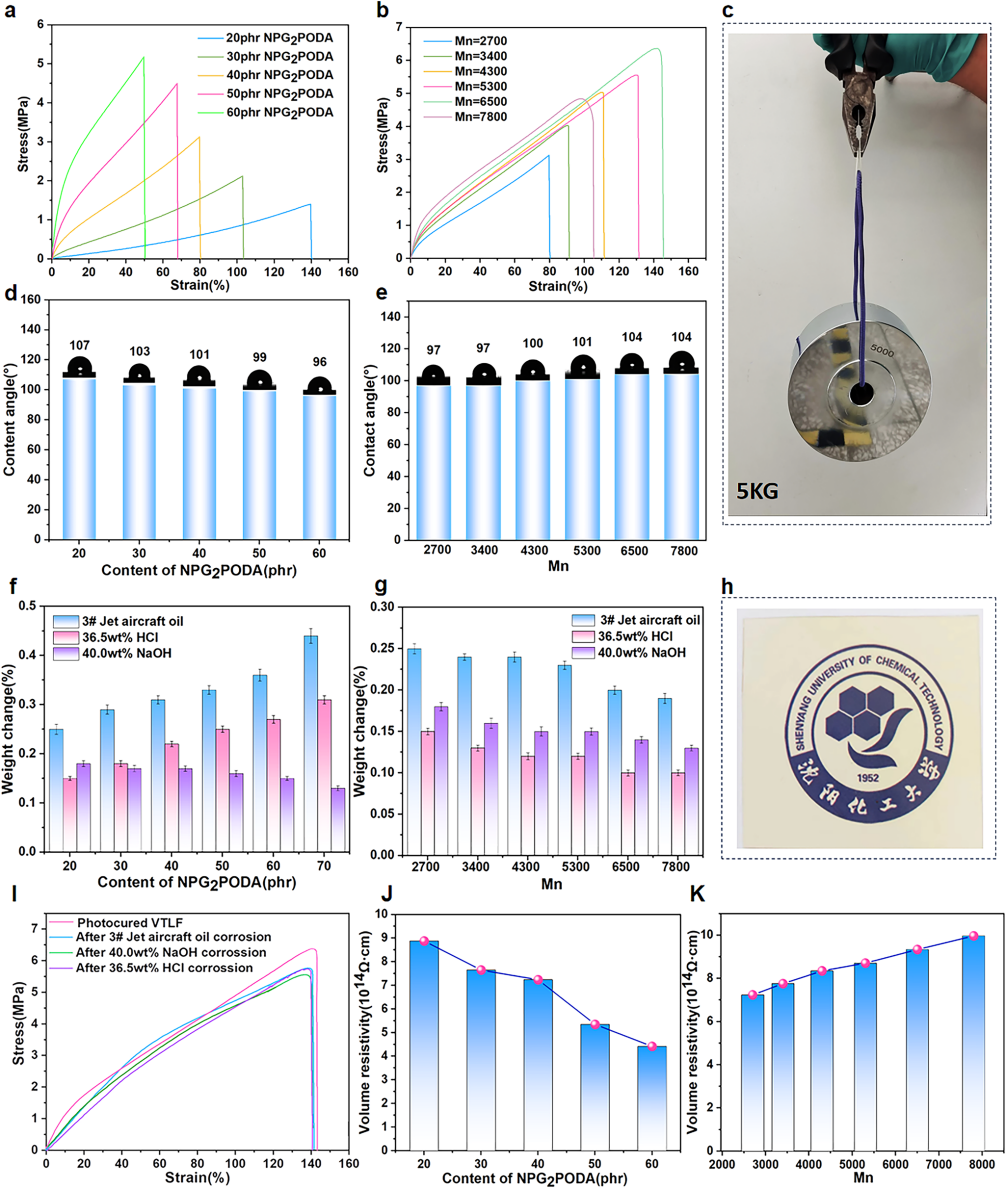
a) Mechanical properties of photocured VTLF with different NPG_2_PODA content. b) Mechanical properties of photocured VTLFs with different Mn. c) The photocured VTLF with a thickness of 0.5 mm lifted a burden of 5 kg. d) Water contact angle of photocured VTLFs with different NPG_2_PODA contents. e) Water contact angle of photocured VTLFs with different Mn. f) Weight changes of photocured VTLFs with different NPG_2_PODA contents in different solvents. g) Weight changes of photocured VTLFs with different Mn in different solvents. h) The maximum light transmittance of the VTLF with a thickness of 0.5 mm is 94.21%. i) Mechanical properties of photocured VTLF after immersion in different solvents. j) Volume resistivity of photocured VTLFs with different NPG_2_PODA content. k) Volume resistivity of photocured VTLF with different Mn.

In terms of the mechanical properties, the stress‒strain curves of the photocured VTLFs with different NPG_2_PODA contents are shown in **Figure** **7**a and their hardnessesare shown in Figure S8 (Supporting Information). When the content of NPG_2_PODA was 20 phr, the tensile strength and elongation at break of the photocured VTLF were only 1.4 MPa and 139%, respectively, with a toughness of 81.9 J m^−1^. It is noteworthy that with increasing NPG_2_PODA content, not only is the crosslinking degree of the photocured product improved, but also, according to the results of the SSRF, a hard segment structure similar to that in polyurethane is formed in the crosslinked network, enhancing the tensile strength of the cured product. Consequently, when the NPG_2_PODA content reached 60 phr, the tensile strength reached 5.2 MPa, though the elongation at break was only 50%. An active diluent content that is too high will also decrease the toughness of cured products. Thus, the optimal content of NPG_2_PODA was 40 phr, and the influence of different molecular weight VTLF on the mechanical properties of the photocured products was investigated. The results are shown in Figures 7b and S9 (Supporting Information).

With an increase in the molecular weight of VTLF, the length of its flexible molecular chain also increases, thereby enhancing the tensile strength and elongation at break of the photocured VTLF. The molecular weight of VTLF was 6500, resulting in a tensile strength of 6.3 MPa, elongation at break of 144%, and toughness of 533.3 J m^−1^. This phenomenon arises because higher molecular weight reduces the content of photocurable vinyl groups, influencing the structure of the crosslinked network. Additionally, excessively long molecular chains of VTLF significantly elevate system viscosity, thereby complicating the molding process. Therefore, when the molecular weight of the VTLF is 7800, the tensile strength and elongation at break of the photocured VTLF are only 4.9 MPa and 100% due to the influence of several unfavorable factors. It can be seen that the performance of the photocured VTLF is best when the molecular weight of the VTLF is 6500, as shown in Figure 7c, and the static and dynamic load‐bearing capacity of the photocured product can easily exceed 5 kg and the maximum light transmittance of 94.21% (Figure 7h).

In terms of surface characteristics, fluoropolymers are distinguished by low surface energy and surface enrichment due to the migration of fluorinated chain segments, resulting in increased fluorine content on the material's surface and high hydrophobicity.^[^ ^40^, ^41^ ^]^ Contact angle measurements were conducted to explore the surface properties of the photocured VTLFs, illustrated in Figure 7d,e. Increasing NPG_2_PODA content reduces the fluorine content in the photocured products, thereby decreasing the contact angle. For instance, at a VTLF molecular weight of 2700 and 20 phr NPG_2_PODA content, the contact angle reached 107°. Conversely, maintaining a constant NPG_2_PODA content while increasing the VTLF molecular weight enhances the fluorine content in the photocured VTLF, promoting greater surface fluorine enrichment and higher contact angles. As expected, the water contact angle of the photocured VTLF could be tailored within the range of 96°–107°. In conclusion, photocured VTLF exhibit excellent hydrophobic properties.

Regarding chemical stability, investigations using 3# Jet aircraft oil, 36.5 wt% HCl, and 40.0 wt% NaOH were conducted to assess the photocured VTLF, with results presented in Figure 7f,g. After 72 h of immersion, the mass change ratios of the photocured VTLF in these highly polar oil, strong acid, and strong alkali solutions were all below 0.45%. This underscores the excellent chemical stability of photocured VTLFs, attributed to the high bond energy and shielding effect of C─F bonds, akin to high‐molecular‐weight fluoropolymers. Notably, photocured VTLFs exhibited superior stability compared to vulcanized fluororubbers using ammonia vulcanizers, which showed a mass change ratio exceeding 2% after 72 h in 40.0 wt% NaOH.^[^ ^42^ ^]^ Additionally, after 72 h of immersion, photocured VTLFs maintained a tensile strength‐to‐fracture elongation ratio above 90% (Figure 7i) and achieved a shape fixation ratio of 98% (Figures S11 and S12, Supporting Information). Specifically, the tensile strength retention rate was 93.33%, and the elongation retention rate was 99.18%. Thus, the results affirm the exceptional chemical stability of photocured VTLF, with highly satisfactory test outcomes.

In terms of optical properties, a level of light transmittance is exhibited by photocured VTLF that is not typically observed in most fluororubbers or fluoroplastics. The UV‒visible spectrum(Figure S10, Supporting Information) shows that the maximum transmittance of the 0.5 mm thick photocured VTLF sheet in the visible wavelength range of 400–800 nm was maintained at 94.21% after 72 h of immersion in various solvents.

In terms of electrical properties, investigations into the volume resistivity and dielectric constant of photocured VTLFs are presented in Figure 7j,k. Due to the high electronegativity of fluorine atoms, the stable C─F bond with carbon atoms prevents electron migration under an electric field. This stability results in high volume resistivity values ranging from 4.42 × 10^14^ to 9.97 × 10^14^ Ω cm. The electrical characteristics of the samples correlate with their surface properties, influenced by NPG_2_PODA content and VTLF molecular weight. Increasing NPG_2_PODA content reduces fluorine content and volume resistivity, reaching a minimum of 4.42 × 10^14^ Ω cm at 60 phr (Figure 7i). Conversely, at a constant NPG_2_PODA content, higher VTLF molecular weights increase fluorine content and volume resistivity, peaking at 9.97 × 10^14^ Ω cm with a molecular weight of 7800 (Figure 7j), accompanied by a dielectric constant of 1.9–2.4 (Figure S13, Supporting Information). In summary, photocured VTLFs exhibit excellent electrical insulation properties.

## 3 Conclusion

In this study, a pioneering oxidative degradation process utilizing multi‐field coupling (microwave, mechanical, steady‐state temperature) was innovatively employed to fabricate photocurable VTLF with high fluorine content and excellent temperature resistance, facilitating efficient recycling of waste fluororubbers. Initially, KOH and H_2_O_2_ systems were used to directly degrade fluororubbers into CTLF with adjustable molecular weights (2300–10400 g mol^−1^). Subsequently, a condensation reaction between carboxyl and hydroxyl groups successfully introduced photosensitive vinyl groups at the chain ends, achieving a conversion rate of up to 93%. These dual reactions could be integrated into a single pot process without intermediate product isolation. Moreover, this method enabled precise control over the molecular weight and end‐group content of telechelic liquid fluororubbers, addressing the challenge of balancing high polarity with low viscosity in fluoropolymers. Subsequent to this, a free radical photocuring system for VTLF was established using NPG_2_PODA as the active diluent and BAPO as the photoinitiator. Exposure to 405 nm UV light resulted in complete crosslinking within 30 s. The photocured VTLFs exhibited hydrophobic properties, with water contact angles ranging from 96° to 107°. These products displayed strong mechanical properties, with a tensile strength up to 6.3 MPa and elongation at break up to 144%. Significantly, these mechanical characteristics were maintained after exposure to 3# Jet aircraft oil, 36.5 wt% HCl, and 40.0 wt% NaOH for 72 h. Moreover, the photocured VTLFs retained a maximum light transmittance of 94.21%, demonstrating excellent chemical stability. Additionally, the photocured VTLFs exhibited effective electrical insulation properties, with volume resistivity ranging from 4.42 to 9.97 × 10^14^ Ω cm. All synthesis steps were conducted under mild conditions, eliminating the need for high temperatures, pressures, or toxic solvents. This study introduces an innovative approach to recycling waste fluororubbers and provides new insights into the efficient synthesis, design of reactive chain structures, and effective curing of functionalized low‐molecular‐weight fluoropolymers.

## 4 Experimental Section

### Preparation of CTLF and VTLF

Waste fluororubbers (50 g, amine vulcanization, Cixi Shixin Rubber Technical Service Co., Ltd. and Shenyang Guide Rubber Products Co., Ltd.) and tetrahydrofuran (THF) (150 mL, Kermel Chemical Reagent) were added to a 500 mL double layer reaction flask and then placed in a microwave reactor with a condensation reflux device. After the waste fluororubbers were completely swollen, BTEAC (0.0165 mol, Damas‐beta), KOH and 30 wt% H_2_O_2_ aqueous solutions were added sequentially at 10 °C and stirred for 12 min, then the mixture was acidified with HCl, and filtered to remove impurities. To this phase, 4‐penten‐1‐ol, DIC, DMAP, and TsOH were sequentially added at room temperature and allowed to react for 30 min. After completion of the reaction, the solution was transferred to water and subjected to multiple washes. The organic phase, recovered from the bottom layer, was subsequently dried under vacuum at 50 °C until reaching a constant weight. If want to individually synthesize CTLF, after subjecting the mixture to microwave irradiation for 12 minutes, it should be subsequently treated with HCl under stirring for 4 h. Subsequently, impurities were removed by filtration and an excess of deionized water was added. The resulting product was dried at 40 °C under vacuum to constant weight.

### Photocuring of VTLF

BAPO was employed as the photoinitiator, while NPG_2_PODA functioned as the active diluent. VTLF, along with BAPO (6 phr) and NPG_2_PODA (40 phr), were blended to create a premix. This mixture was then uniformly poured into a glass mold and exposed to 405 nm UV light for 30 s to achieve the photocured VTLF.

### Characterization

FT‐IR spectroscopy was performed using a Thermo Fisher Scientific Nicolet is10 infrared spectrometer with a scan range of 400–4000 cm^−1^ over 16 scans. Acetone was used as the solvent for the qualitative analysis of the structure. ^1^H‐NMR spectroscopy was performed using an AVANCE‐III‐500 MHz spectrometer (Bruker, Switzerland) with C_3_D_6_O as the solvent and TMA as the standard. ^19^F‐NMR spectra were obtained using an AVANCE‐NEO‐400 MHz spectrometer (Bruker, Switzerland), and the standard was CFCl_3_. The Mn of the fluororubbers was determined using a GPC system (PL‐GPC50) from Varian, Inc. PS served as the mobile phase at a flow rate of 1 mL min^−1^ and a test temperature of 30 °C. DSC measurements were conducted using a TA thermal analyzer (USA) under nitrogen atmosphere, with a constant temperature of 40 °C. The testing involved a temperature range of ‐100 to +30 °C, with a ramp‐up rate of 10 °C min^−1^, to assess the glass transition temperature (*T*_g_) of the products. Mechanical properties were evaluated according to GB/T 528‐2009 standard using an Instron 3365 universal tensile testing machine. At a test temperature of 25 °C, at least 5 samples were tested at a tensile rate of 50 mm min^−1^, and the average value was calculated. The transmittance of photocured products was tested under UV light in the wavelength range of 400–800 nm using a Hitachi uH5700 UV–visible near‐infrared spectrophotometer from Japan. Static contact‐angle tests were performed using a Dataphysics OCA20 instrument (SINDIN SDC100, Dataphysics, Germany) with a test water volume of 1 µL. Five points on each sample were selected and tested, and the average was taken as the WCA of the sample. Synchrotron radiation measurements were carried out on fluororubbers and photocured VTLF at the BL16B1 beamline of the Shanghai Synchrotron Radiation Facility (SSRF, Shanghai, China). Samples were loaded into a sample cell coated with Kapton film. The energy and wavelength of the X‐ray were set to 10 keV and 1.24 Å, respectively, with a camera length of 1950 mm and an accumulation time of 1 s. The beam size at the sample position was 0.39 × 0.48 mm^2^. The scattering intensity for each sample was measured using a Dectris PILATUS 2MF instrument (DECTRIS, Baden, Switzerland). SG‐Tools software was used for image corrections, background and transmission effects, data reduction, and radial integration of the 2D patterns to export 1D synchrotron radiation profiles.

## Conflict of Interest

The authors declare no conflict of interest.

## Author Contributions

D.L. and L.Y. contributed equally to this work. D.L., L.Y., S.N., and P.L. supervised the study. D.L., L.Y., S.N., P.L., C.C., D.Z., S.Z., M.L., and Q.M. performed the experiment. D.L., Q.F., H.K., L.L., and J.Y. performed the acquisition and analysis of the data and statistical analysis. All the authors drafted the manuscript. D.L., L.Y., C.C., D.Z., M.L., and Q.M. revised the manuscript. All the authors have read and approved the final version of the manuscript.

## Supporting information



Supporting Information

## Data Availability

The data that support the findings of this study are available in the Supporting Information of this article.
