# Supplementary material for: Upcycling of Waste Fluororubber to Photocurable High‐Performance Vinyl‐Terminated Liquid Fluororubber by Multifield Coupling One‐Pot Stepwise Reactions
Source: Adv Sci (Weinh). 2025 May 28;12(31):e01460. doi: 10.1002/advs.202501460 (PMC12376610; doi:10.1002/advs.202501460)
Supplement: Supplementary file 1 — Supporting Information [file ADVS-12-e01460-s001.docx]

**Supporting Information**

**Upcycling of waste fluororubber to photocurable high-performance vinyl-terminated liquid fluororubber by multi-field coupling one-pot step-wise reactions**

Donghan Li, ^*,#^ Lu Yu ^#^, Shurui Ning, Ping Li, Changle Chen ^*^, Dawei Zhao, Mingyi Liao, Qingshi Meng, Shixin Zhang , Qinghong Fang , Hailan Kang , Long Li , Jia Yang^*^


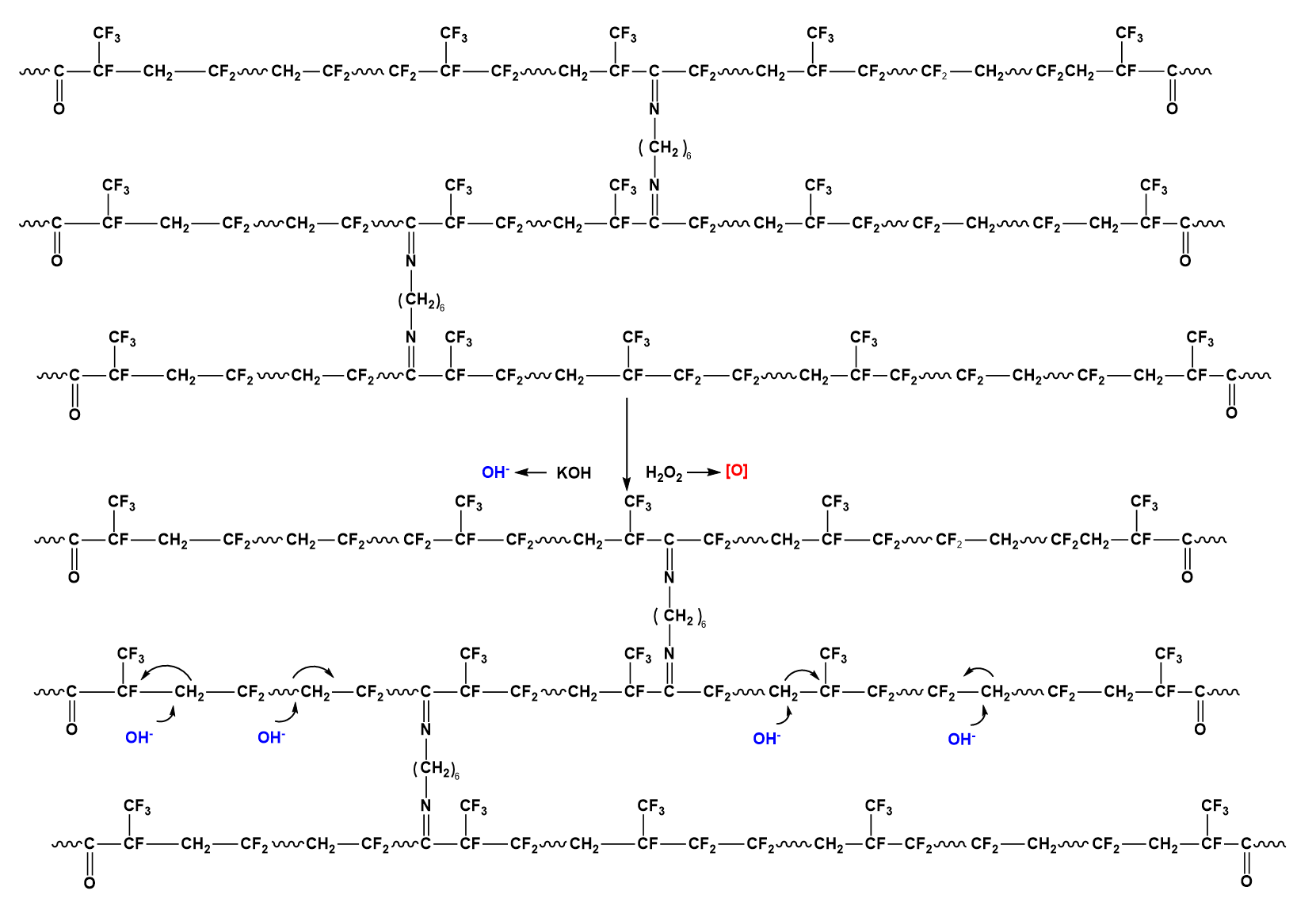


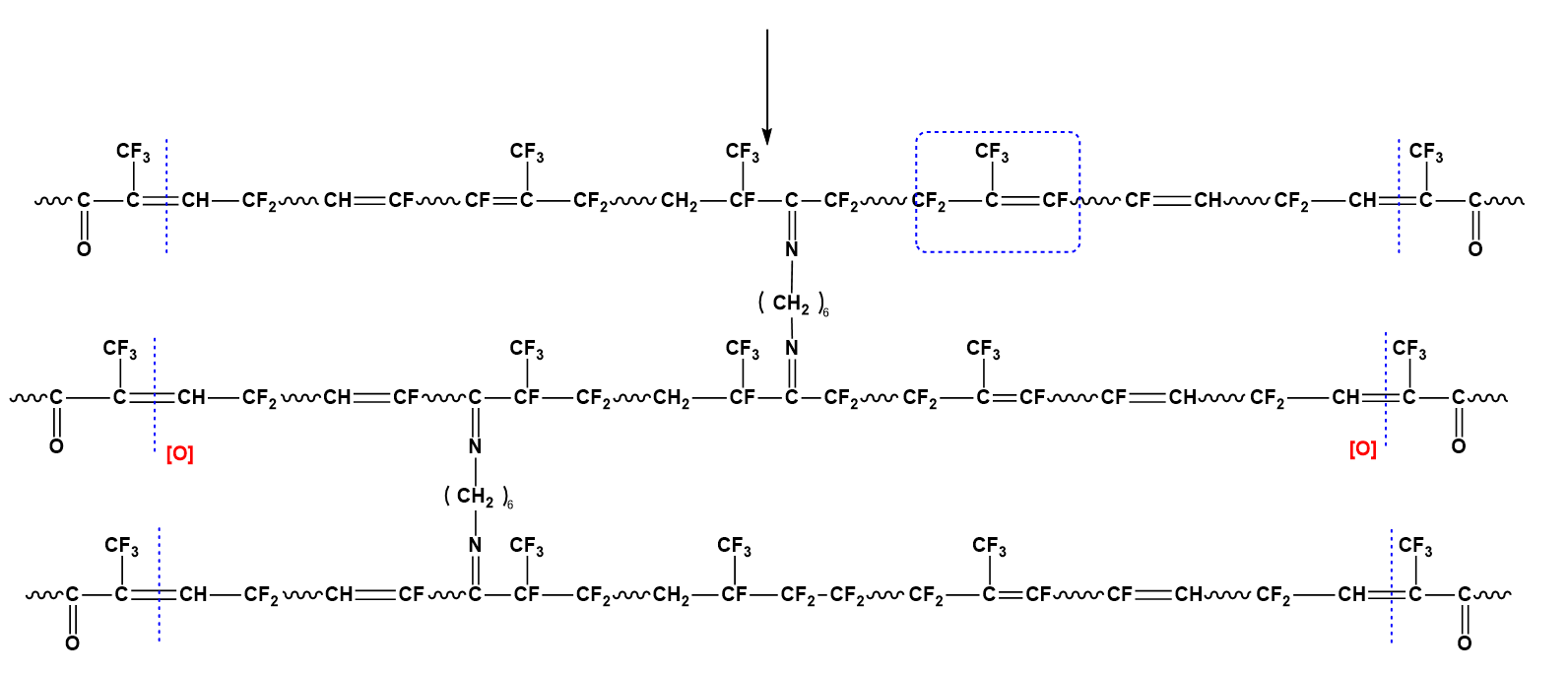


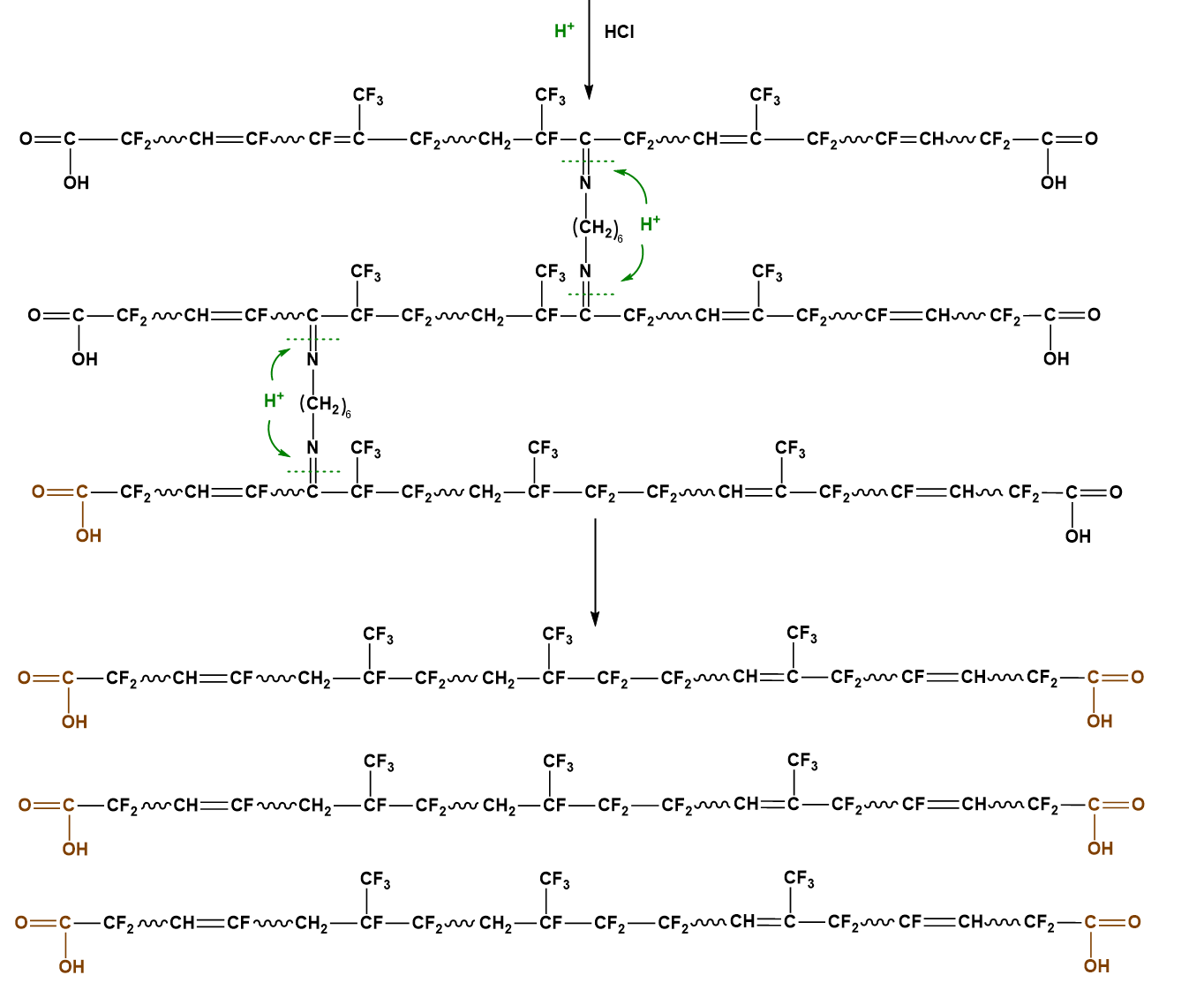


**Figure S1.** Oxidative degradation reaction mechanism of waste fluororubbers.


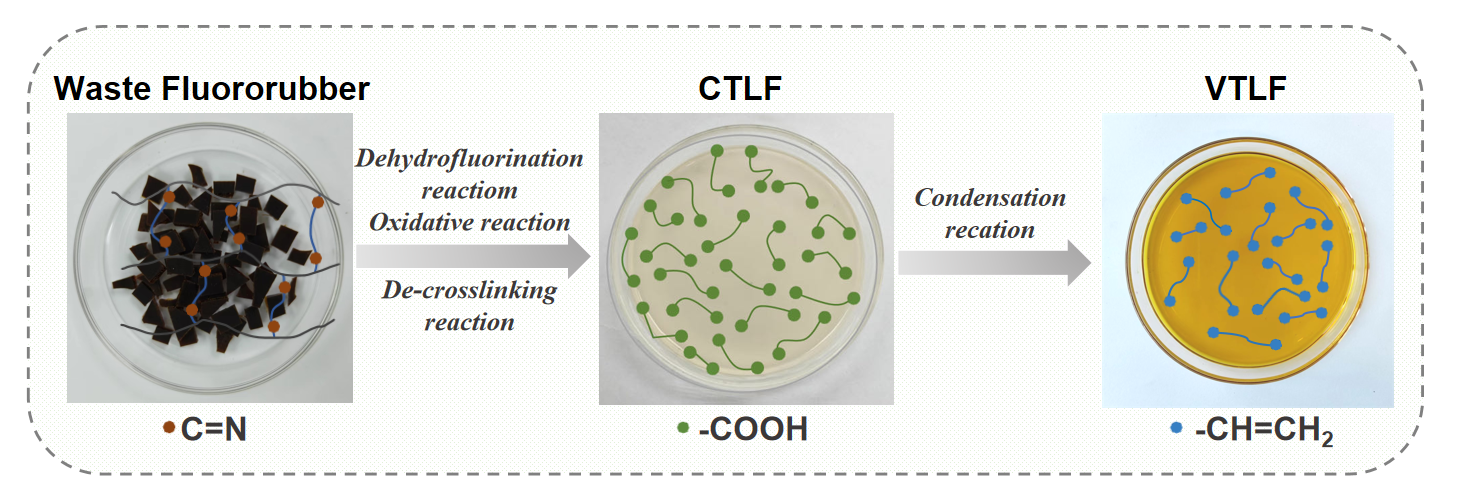


**Figure S2.** Process of upcycling waste fluororubbers into high-performance liquid fluororubbers.


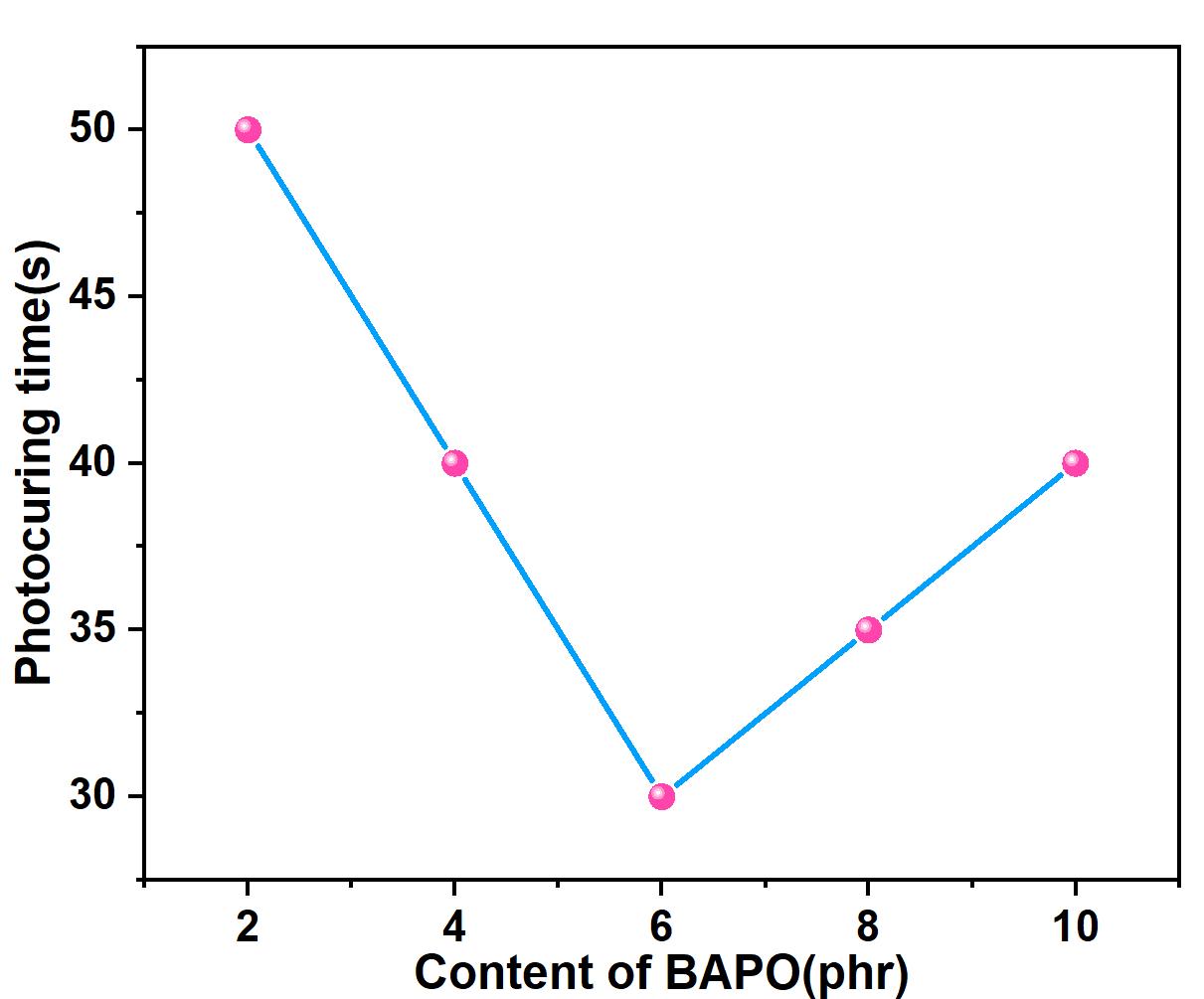


**Figure S3.** Effect of the amount of BAPO on the photocuring time.

**
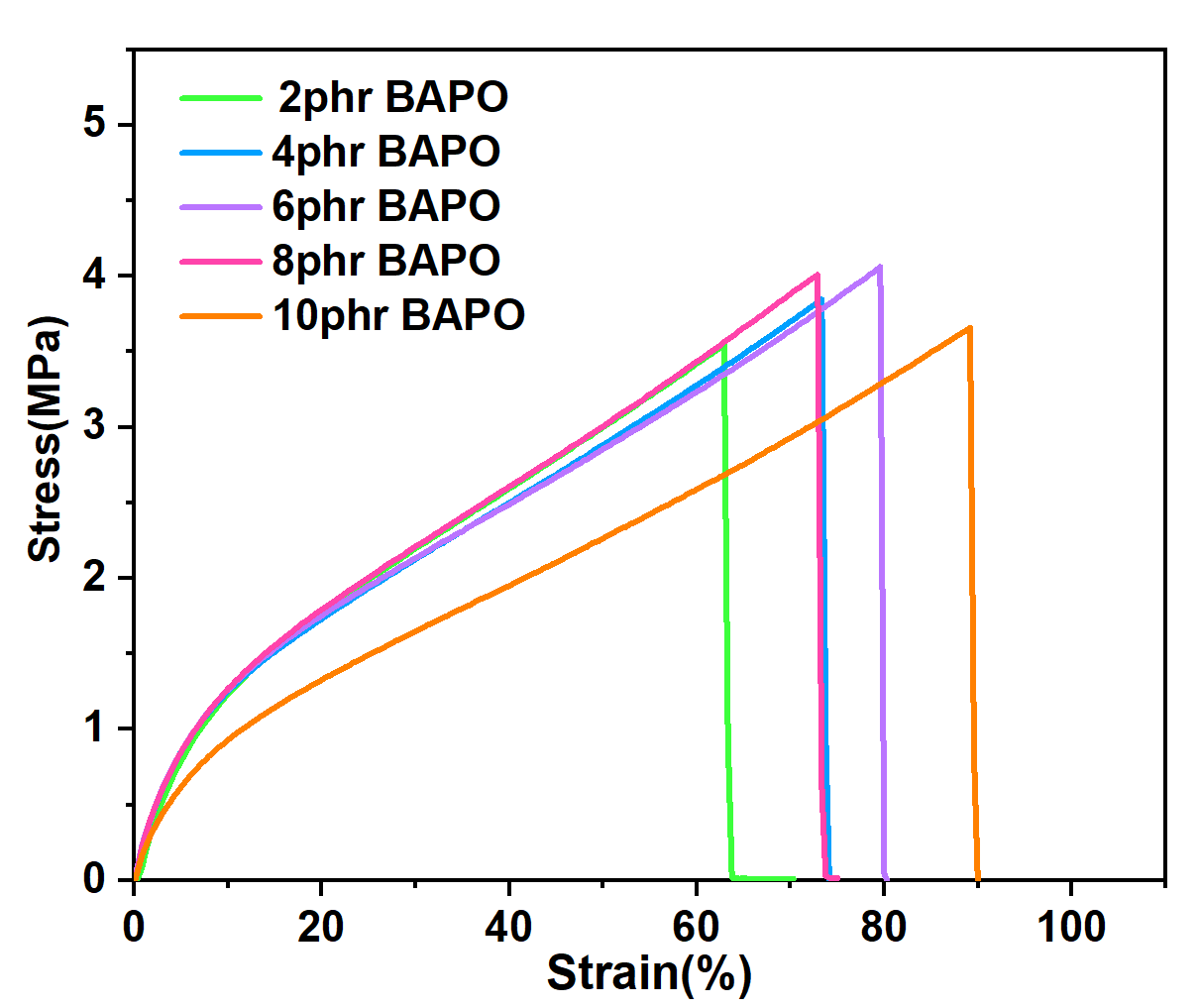
**

**Figure S4.** Effect of the amount of BAPO on mechanical properties of photocured VTLFs.


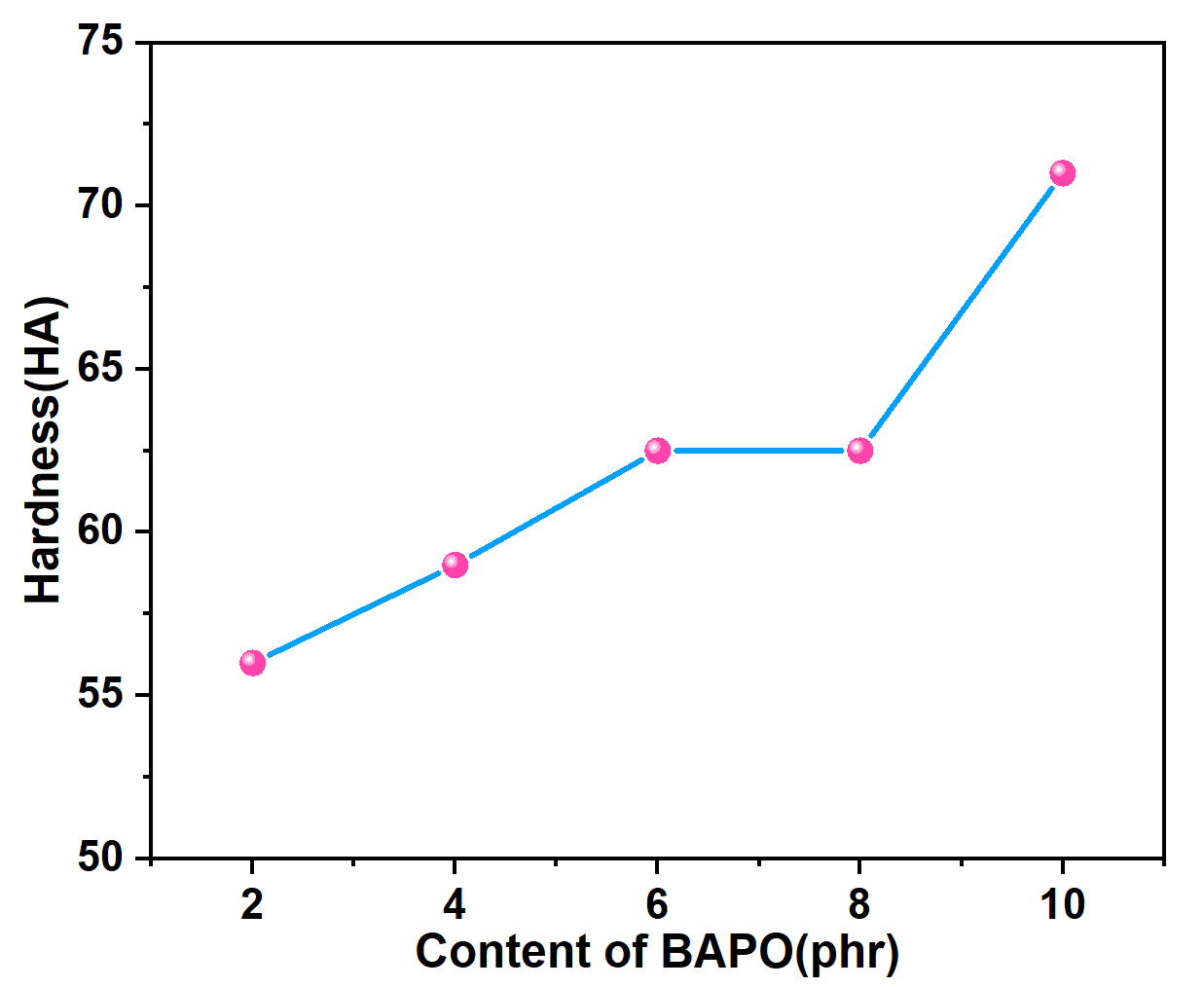


**Figure S5.** Effect of the amount of BAPO on the hardness of photocured VTLFs.


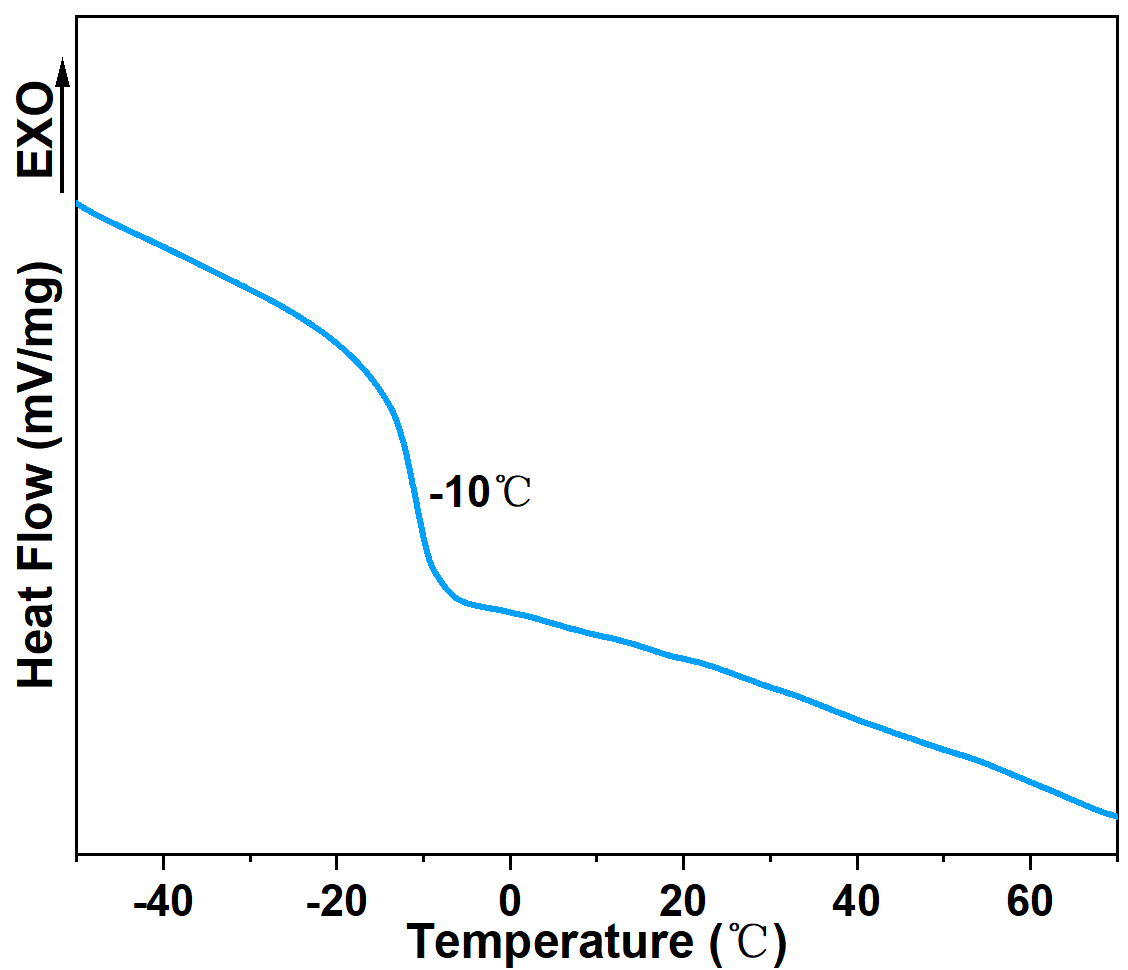


**Figure S6.** DSC curve of intact fluororubber.


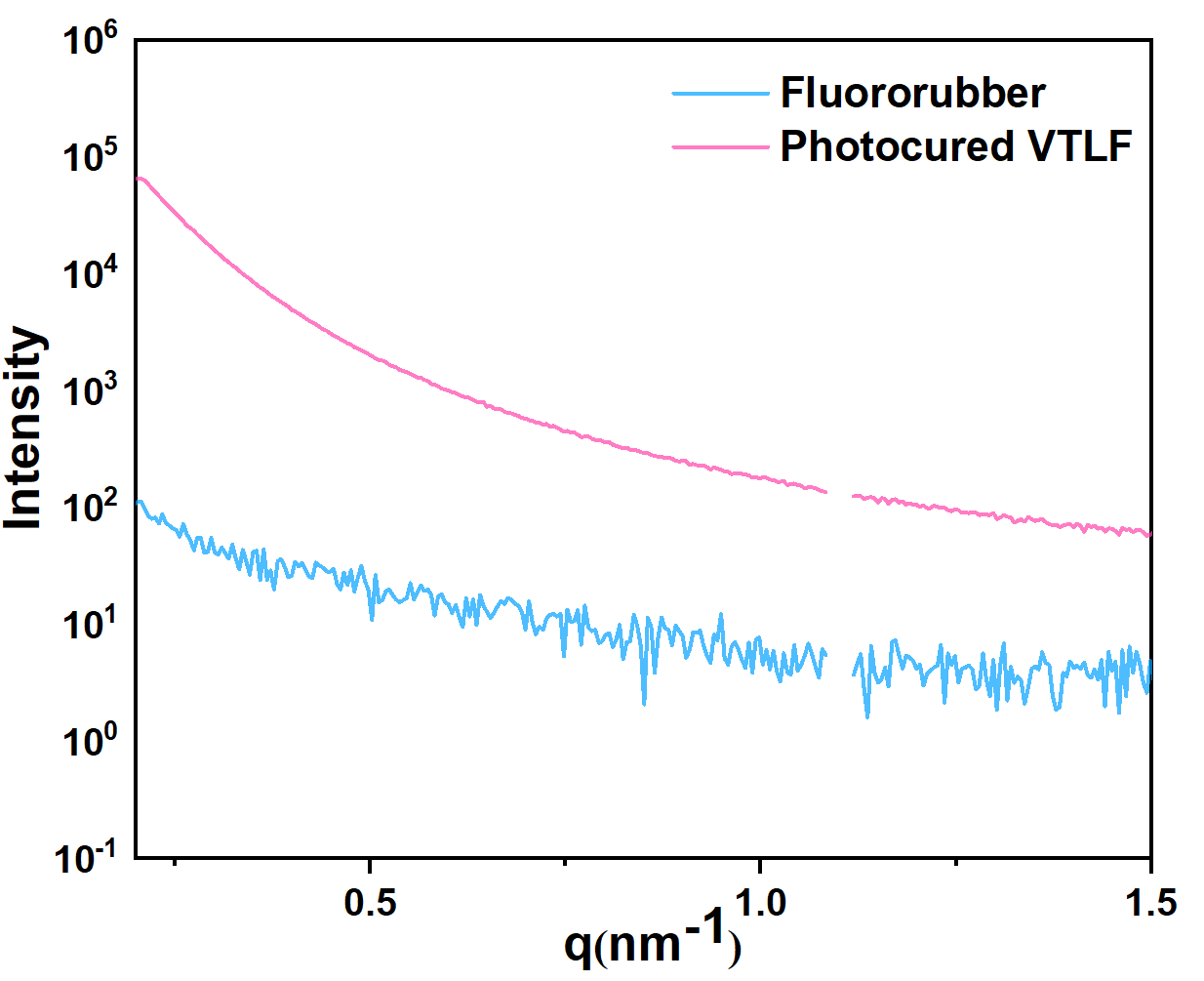


**Figure S7.** The 1D SAXS profiles of the fluororubber and photocured VTLF were exported from SG-Tools.


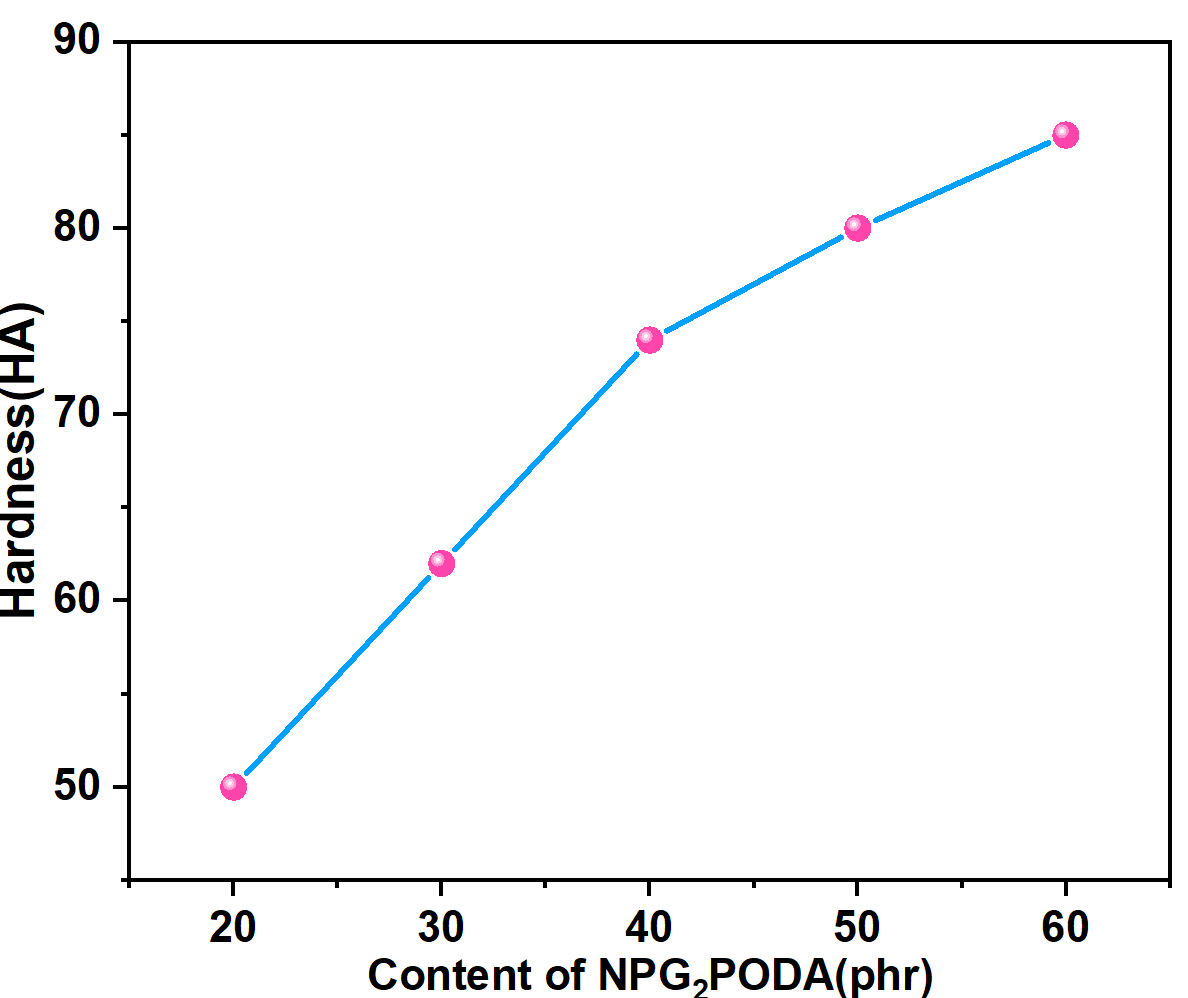


**Figure S8.** Effect of NPG_2_PODA content on the hardness of photocured VTLFs.


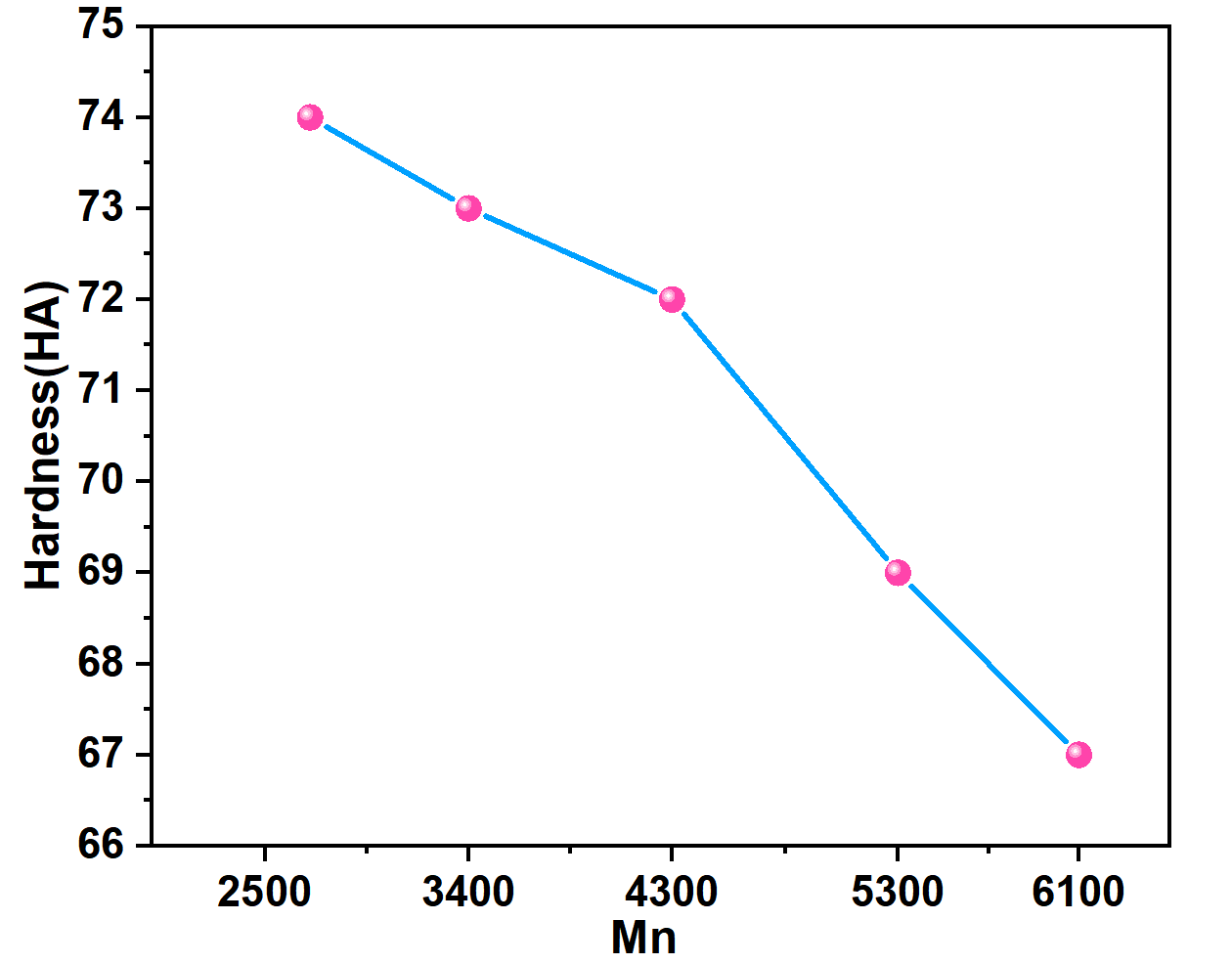


**Figure S9.** Effect of molecular weight on hardness of photocured VTLFs


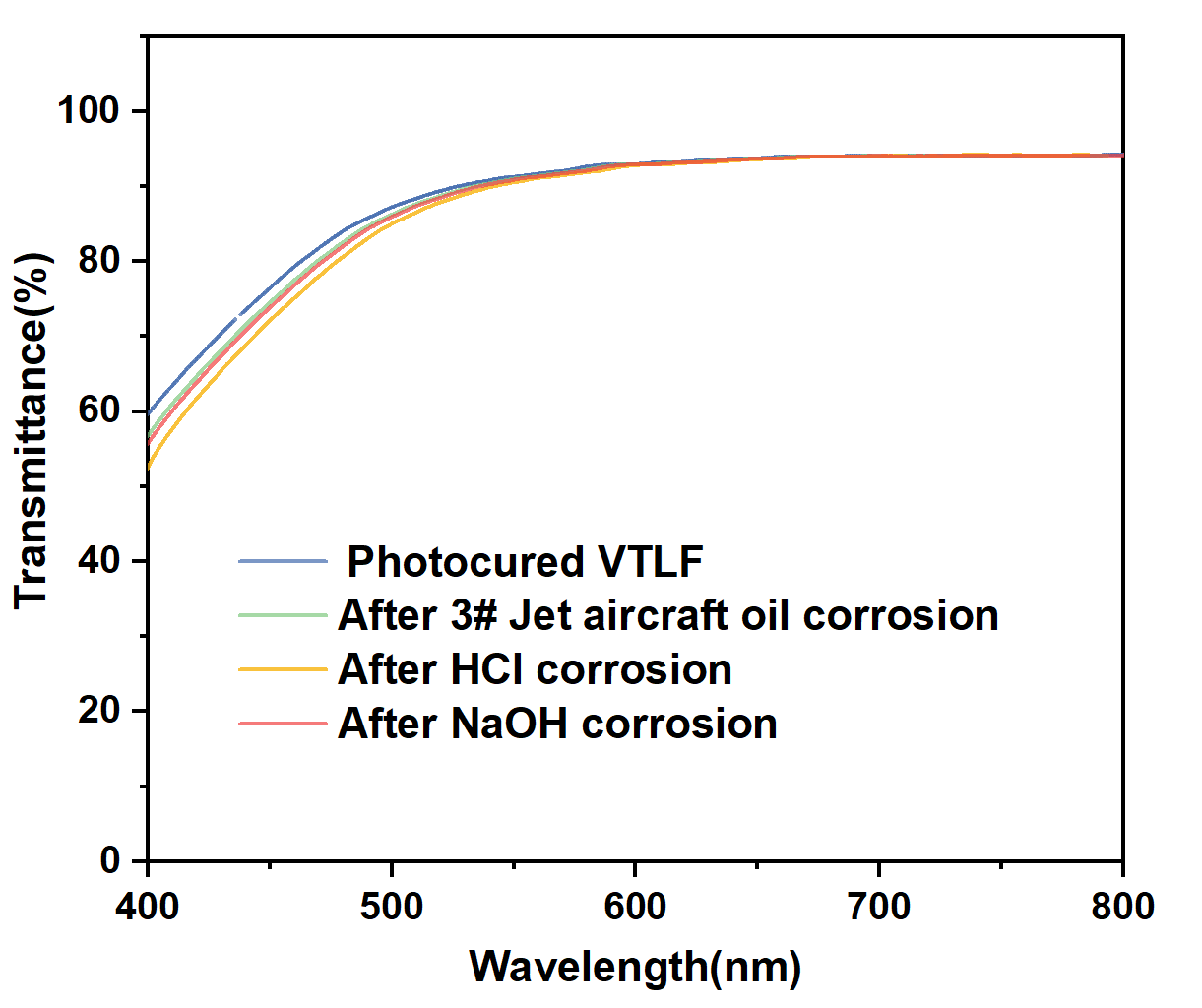


**Figure S10.** The transmittance of photocured VTLF with a thickness of 0.5 mm at the wavelength of 400 nm to 800 nm before and after 72h corrosion with different solvents.


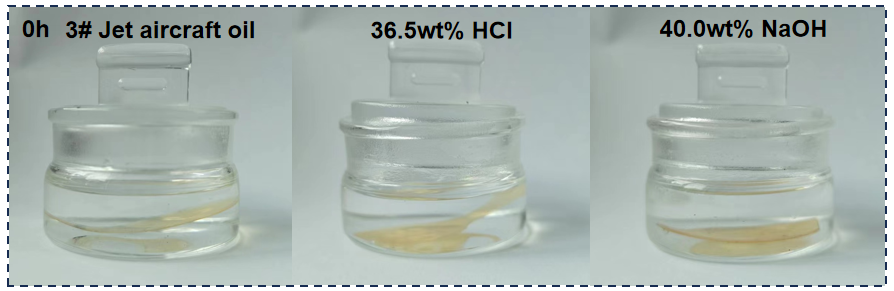


**Figure S11.** Pictures of photocured VTLF in different solvents for 0h.


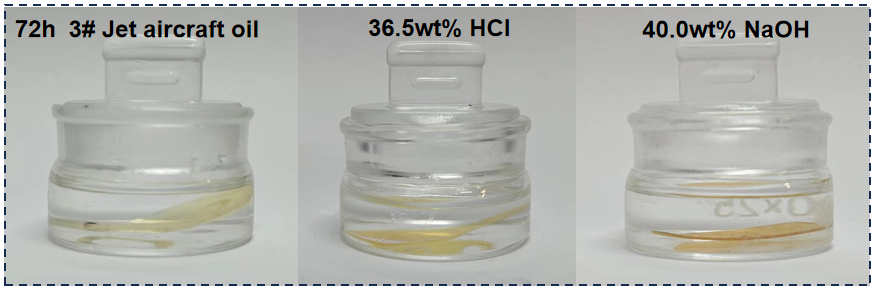


**Figure S12.** Pictures of photocured VTLF in different solvents for 72h.

**
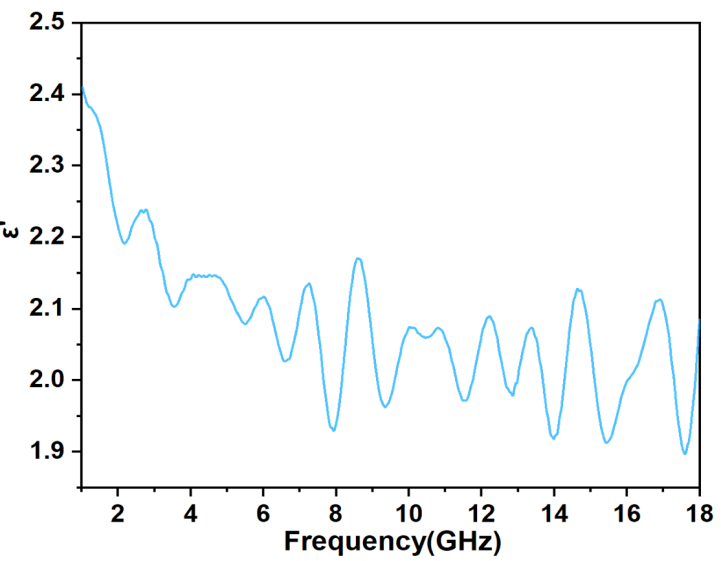
**

**Figure S13.** Dielectric constant of photocured VTLF with Mn is 7800.

**Table S1.** The composition of waste fluororubber

| Composition | Fluororubber | Stabilizing agent | Metal oxidizer | Vulcanizing agent |
| --- | --- | --- | --- | --- |
|  | Poly (VDF-co-HFP)copolymer | Barium sulfate | Magnesium oxide | N, N-Dicinnamylidene-1,6-hexanediamine |

**Table S2**. Assignments of ^19^F-NMR peaks in CTLF

| δ(ppm) | Assignments | δ(ppm) | Assignments |
| --- | --- | --- | --- |
| -64.30 | –CF_2_C***F_2_***COOH | -104.31 | –CF_2_CH_2_C***F***_2_CF(CF_3_)CF_2_– |
| -71.35 | –CH_2_CF_2_CF(C***F_3_***)CF_2_CH_2_– | -109.69 | –CF(CF_3_)CH_2_C***F***_2_CF(CF_3_)CF_2_– |
| -74.85 | –CF_2_CH=C(C***F_3_***)CF_2_– | -111.27 | –CF_2_CH_2_C***F***_2_CF_2_CF(CF_3_)– |
| -75.88 | –CF_2_CH_2_CF(C***F_3_***)CF_2_CF_2_– | -113.29 | –CF(CF_3_)CH_2_C***F***_2_CF_2_CH_2_– |
| -81.24 | –CH=CFCF(C***F_3_***)– | -114.78 | –CF_2_CH_2_C***F***_2_CF_2_CH_2_– |
| -81.48 | –CF=CHCF(C***F_3_***)CF_2_– | -116.40 | –CF=CHCF(C***F_3_***)CF_2_– |
| -92.36 | –CF_2_CH_2_C***F***_2_CH_2_CF_2_– | -116.24 | –CH_2_CH_2_C***F***_2_CF_2_CF(CF_3_)– |
| -94.38 | –CF_2_CH_2_C***F***_2_CH_2_CF(CF_3_)– | -119.43 | –CH_2_CF_2_C***F***_2_CF(CF_3_)CH_2_– |
| -96.14 | –CH_2_CH_2_C***F***_2_CH_2_CF_2_– |  |  |

**Table S3**. Effect of microwave power on the characteristics of CTLFs

| Microwave(W) | COOH (wt%) | M_n_^a^ | PDI |
| --- | --- | --- | --- |
| 80 | 2.39 | 3100 | 2.32 |
| 160 | 2.72 | 2300 | 1.78 |
| 240 | 2.82 | 2100 | 1.79 |
| 320 | 2.70 | 2100 | 1.84 |
| 400 | 2.62 | 2300 | 1.79 |
| 480 | 2.55 | 2600 | 1.79 |

a:The molecular weights in the table have been rounded to the nearest whole number.

**Table S4**. Effect of microwave radiation time on the characteristics of CTLFs

| Time(min) | COOH (wt%) | M_n_^a^ | PDI |
| --- | --- | --- | --- |
| 6 | 2.32 | 2600 | 1.88 |
| 12 | 2.47 | 2500 | 1.72 |
| 18 | 2.68 | 2300 | 1.80 |
| 24 | 2.82 | 2100 | 1.79 |
| 30 | 2.75 | 2500 | 1.89 |

a:The molecular weights in the table have been rounded to the nearest whole number.

**Table S5**. Effect of H_2_O_2_/KOH molar ratio on the characteristics of CTLFs

| H_2_O_2_/KOH molar ratio | COOH (wt%) | M_n_^a^ | PDI |
| --- | --- | --- | --- |
| 0.7/1.0 | 2.15 | 3300 | 2.15 |
| 0.8/1.0 | 2.22 | 2800 | 1.95 |
| 0.9/1.0 | 2.56 | 2600 | 1.88 |
| 1.0/1.0 | 2.82 | 2100 | 1.79 |
| 1.1/1.0 | 3.25 | 2000 | 1.73 |

a:The molecular weights in the table have been rounded to the nearest whole number.

**Table S6**. Effect of microwave power on the characteristics of VTLFs

| Microwave(W) | M_n_^a^ | PDI | Conversion rate (α%) | C=C(wt%) |
| --- | --- | --- | --- | --- |
| 160 | 2600 | 1.80 | 54 | 1.33 |
| 240 | 2700 | 1.76 | 78 | 1.92 |
| 320 | 2700 | 1.80 | 93 | 2.30 |
| 400 | 3000 | 1.81 | 87 | 2.15 |
| 480 | 2900 | 1.84 | 85 | 2.10 |

a:The molecular weights in the table have been rounded to the nearest whole number.

**Table S7**. Effect of microwave radiation time on the characteristics of VTLFs

| Time(min) | M_n_^a^ | PDI | Conversion rate (α%) | C=C(wt%) |
| --- | --- | --- | --- | --- |
| 10 | 2600 | 1.78 | 56 | 1.38 |
| 20 | 2800 | 1.84 | 85 | 1.73 |
| 30 | 2700 | 1.80 | 93 | 2.30 |
| 40 | 2700 | 1.79 | 88 | 2.17 |
| 50 | 2700 | 1.83 | 85 | 2.10 |

a:The molecular weights in the table have been rounded to the nearest whole number.

**Table S8**. Effect of 4-penten-1-ol dosages on the characteristics of VTLFs

| COOH/4-penten-1-ol molar ratio | M_n_^a^ | PDI | Conversion rate (α%) | C=C(wt%) |
| --- | --- | --- | --- | --- |
| 1.0/0.5 | 2600 | 2.40 | 66 | 1.63 |
| 1.0/1.0 | 2600 | 2.01 | 83 | 2.05 |
| 1.0/1.5 | 2800 | 1.98 | 84 | 2.07 |
| 1.0/2.0 | 2700 | 1.80 | 93 | 2.30 |
| 1.0/2.5 | 2900 | 1.89 | 88 | 2.17 |
| 1.0/3.0 | 2800 | 2.07 | 80 | 1.98 |

a:The molecular weights in the table have been rounded to the nearest whole number.

**Table S9**. Effect of DIC dosages on the characteristics of VTLFs

| COOH/DIC molar ratio | M_n_^a^ | PDI | Conversion rate (α%) | C=C(wt%) |
| --- | --- | --- | --- | --- |
| 1.0/0.6 | 2700 | 2.09 | 63 | 1.55 |
| 1.0/0.8 | 2800 | 1.98 | 86 | 2.12 |
| 1.0/1.0 | 2700 | 1.80 | 93 | 2.30 |
| 1.0/1.2 | 2800 | 1.79 | 85 | 2.10 |
| 1.0/1.4 | 2800 | 1.77 | 82 | 2.02 |
| 1.0/1.6 | 2800 | 1.81 | 84 | 2.07 |

a:The molecular weights in the table have been rounded to the nearest whole number.

**Table S10**. Effect of DMAP dosages on the characteristics of VTLFs

| COOH/DMAP molar ratio | M_n_^a^ | PDI | Conversion rate (α%) | C=C(wt%) |
| --- | --- | --- | --- | --- |
| 1.0/0.09 | 2500 | 1.86 | 46 | 1.14 |
| 1.0/0.11 | 2600 | 1.91 | 66 | 1.63 |
| 1.0/0.13 | 2700 | 1.84 | 83 | 2.05 |
| 1.0/0.15 | 2700 | 1.80 | 93 | 2.30 |
| 1.0/0.17 | 2800 | 2.00 | 85 | 2.10 |
| 1.0/0.19 | 2700 | 2.03 | 81 | 2.00 |

a:The molecular weights in the table have been rounded to the nearest whole number.

**Table S11**. Effect of TsOH dosages on the characteristics of VTLFs

| COOH/TsOH molar ratio | M_n_^a^ | PDI | Conversion rate (α%) | C=C(wt%) |
| --- | --- | --- | --- | --- |
| 1.0/0.04 | 2600 | 1.96 | 40 | 0.99 |
| 1.0/0.06 | 2700 | 1.87 | 58 | 1.43 |
| 1.0/0.08 | 2800 | 1.95 | 79 | 1.95 |
| 1.0/0.1 | 2700 | 1.80 | 93 | 2.30 |
| 1.0/0.12 | 2800 | 2.06 | 92 | 2.27 |
| 1.0/0.14 | 2700 | 1.81 | 93 | 2.30 |

a:The molecular weights in the table have been rounded to the nearest whole number.
